# A Multi-Center Study Investigating Long COVID-19 in Healthcare Workers from North-Eastern Italy: Prevalence, Risk Factors and the Impact of Pre-Existing Humoral Immunity—ORCHESTRA Project

**DOI:** 10.3390/vaccines11121769

**Published:** 2023-11-27

**Authors:** Luca Cegolon, Marcella Mauro, Donatella Sansone, Alice Tassinari, Fabrizio Maria Gobba, Alberto Modenese, Loretta Casolari, Filippo Liviero, Sofia Pavanello, Maria Luisa Scapellato, Francesco Taus, Angela Carta, Gianluca Spiteri, Maria Grazia Lourdes Monaco, Stefano Porru, Francesca Larese Filon

**Affiliations:** 1 Occupational Medicine Unit, Department of Medical, Surgical & Health Sciences, University of Trieste, 34129 Trieste, Italy; mmauro@units.it (M.M.); donatella.sansone@asugi.sanita.fvg.it (D.S.); alice.tassinari@studenti.units.it (A.T.); larese@units.it (F.L.F.); 2 Occupational Medicine Unit, University Health Agency Giuliano-Isontina (ASUGI), 34129 Trieste, Italy; 3 Department of Biomedical, Metabolic and Neurological Sciences, University of Modena and Reggio-Emilia, 41125 Modena, Italy; fabriziomaria.gobba@unimore.it (F.M.G.); alberto.modenese@unimore.it (A.M.); 4 Health Surveillance Service, Modena University Hospital, 41125 Modena, Italy; l.casolari@ausl.mo.it; 5 Occupational Medicine Unit, Padua University Hospital, 35128 Padua, Italy; filippo.liviero@unipd.it (F.L.); sofia.pavanello@unipd.it (S.P.);; 6 Department of Cardiac, Thoracic and Vascular Sciences, University of Padua, 35128 Padua, Italy; 7 Department of Diagnostics and Public Health, Section of Medical Statistics, University of Verona, 37134 Verona, Italy; francesco.taus@univr.it; 8 Occupational Medicine Unit, Verona University Hospital, 37134 Verona, Italy; angela.carta@univr.it (A.C.); gianluca.spiteri@aovr.veneto.it (G.S.); mariagrazialourdes.monaco@aovr.veneto.it (M.G.L.M.); stefano.porru@univr.it (S.P.); 9 Department of Diagnostics and Public Health, Section of Occupational Medicine, University of Verona, 37134 Verona, Italy

**Keywords:** long COVID-19, SARS-CoV-2, post-infective symptoms, asthenia, pandemic waves, viral shedding time, disease severity, COVID-19 vaccination, humoral immunity

## Abstract

**Introduction:** The impact of long-COVID-19 syndrome is rather variable, since it is influenced by several residual confounders. This study aimed to investigate the prevalence of long COVID-19 in healthcare workers (HCWs) from four university hospitals in north-eastern Italy: Trieste, Padua, Verona, and Modena-Reggio Emilia. **Methods:** During the period June 2022–August 2022, HCWs were surveyed for past COVID-19 infections, medical history, and any acute as well as post-COVID-19 symptoms. The prevalence of long COVID-19 was estimated at 30–60 days or 61+ days since first negative swab following first and second COVID-19 episode. Furthermore, the risk of long COVID-19 was investigated by multivariable logistic regression. Results were expressed as the adjusted odds ratio (aOR) with a 95% confidence interval (95%CI). **Results:** 5432 HCWs returned a usable questionnaire: 2401 were infected with SARS-CoV-2 at least once, 230 were infected at least twice, and 8 were infected three times. The prevalence of long COVID-19 after a primary COVID-19 infection was 24.0% at 30–60 days versus 16.3% at 61+ days, and 10.5% against 5.5% after the second SARS-CoV-2 event. The most frequent symptoms after a first COVID-19 event were asthenia (30.3%), followed by myalgia (13.7%), cough (12.4%), dyspnea (10.2%), concentration deficit (8.1%), headache (7.3%), and anosmia (6.5%), in decreasing order of prevalence. The risk of long COVID-19 at 30–60 days was significantly higher in HCWs hospitalized for COVID-19 (aOR = 3.34; 95%CI: 1.62; 6.89), those infected with SARS-CoV-2 during the early pandemic waves—namely the Wuhan (aOR = 2.16; 95%CI: 1.14; 4.09) or Alpha (aOR= 2.05; 95%CI: 1.25; 3.38) transmission periods—and progressively increasing with viral shedding time (VST), especially 15+ days (aOR = 3.20; 95%CI: 2.07; 4.94). Further determinants of long COVID-19 at 30–60 days since primary COVID-19 event were female sex (aOR = 1.91; 95%CI: 1.30; 2.80), age >40 years, abnormal BMI, or administrative services (reference category). In contrast, HCWs vaccinated with two doses before their primary infection (aOR = 0.57; 95%CI: 0.34; 0.94), undergraduate students, or postgraduate medical trainees were less likely to experience long COVID-19 at 30–60 days. Apart from pandemic waves, the main determinants of long COVID-19 at 30–60 days were confirmed at 61+ days. **Conclusions:** The risk of long COVID-19 following primary infection increased with the severity of acute disease and VST, especially during the initial pandemic waves, when more virulent viral strains were circulating, and susceptibility to SARS-CoV-2 was higher since most HCWs had not been infected yet, COVID-19 vaccines were still not available, and/or vaccination coverage was still building up. The risk of long COVID-19 therefore decreased inversely with humoral immunity at the individual level. Nevertheless, the prevalence of long COVID-19 was remarkably lower after SARS-CoV-2 reinfections regardless of vaccination status, suggesting that hybrid humoral immunity did not increase protection against the syndrome compared to immunity mounted by either natural infection or vaccination separately. Since the risk of long COVID-19 is currently low with Omicron and patients who developed the syndrome following SARS-CoV-2 infection in the early pandemic waves tend to return to a state of full health with time, a cost-effective approach to screen post-COVID-19 symptoms during the Omicron time could be restricted to vulnerable individuals developing severe disease and/or with prolonged VST.

## 1. Introduction

The term “*Long COVID*” was coined by a professor of infectious disease sharing his experience of 7 weeks on a “rollercoaster of ill health” after COVID-19 [1]. Long-COVID-19 syndrome then gained rapidly growing popularity following the publication of several scientific articles covering the topic [2]

The five phases of an infectious disease are: (1) incubation; (2) prodromal; (3) illness; (4) decline; and (5) convalescence. It is therefore expected that patients recovering from a respiratory viral disease may undergo a variable period of convalescence, depending on several factors, especially the virulence of the pathogen and individuals’ susceptibility [3].

Several terms have been proposed thereafter to define the long-term effects of COVID-19 in a proportion of surviving patients: long COVID-19; COVID sequelae; post COVID symptoms; or post-COVID condition; among others [4].

In 2021, the World Health Organization (WHO) defined long COVID-19 as a condition impacting everyday life, lasting at least 2 months, affecting individuals with a history of probable or confirmed SAS-CoV-2 infection, not otherwise explicable, still present 12 weeks after symptom onset and presenting in individuals with a history of probable or confirmed SARS-CoV-2 infection [5]. Although there is still no consensus on post-COVID-19 syndrome, the above WHO definition has been subsequently changed by the USA Center for Disease Control and Prevention (CDC) and the National Institute of Care & Health Excellence (NICE) of the UK, shortening the follow-up time of symptoms to at least 4 weeks since SARS-CoV-2 infection [6,7,8].

The impact of long-COVID-19 syndrome is reportedly very variable since different definitions of long COVID-19 are employed [9,10]. Furthermore, since the diagnosis of long COVID-19 mainly relies on self-reporting symptoms, reporting bias is part of the game, especially in the current society governed by social media, where the public opinion can be easily influenced and/or manipulated or misinformed [11].

The prevalence of long COVID-19 is potentially influenced by several factors, including the study population, the pandemic period of SARS-CoV-2 infection, and the degree of disease severity [10,12,13,14,15].

In an outpatient study on the general population of Trieste, the rate of patients accessing the post-COVID-19 clinic due to symptoms that developed after a median time of 49 days since COVID-19 diagnosis was 1.2% out of a total of 22,073 SARS-CoV-2 infections diagnosed in the entire catchment area between 1 October 2020 and 2 March 2021 [16]. The risk of long COVID-19 has progressively diminished over time over the course of the pandemic, especially with the spread of Omicron, a highly contagious yet less virulent variant [12].

Most studies in the open literature have attempted to estimate long COVID-19 in the general population, which is quite heterogeneous in terms of COVID-19 vaccine uptake and access to SARS-CoV-2 swab diagnostic tests [13]. From this point of view, healthcare workers (HCWs) are a particular sub-group that are relatively more homogeneous for various characteristics, including a higher biological risk combined with enhanced COVID-19 vaccine coverage compared to the general population. HCWs were also subject to a systematic yet mandatory screening schedule against SARS-CoV-2 infection since the start of the pandemic [17,18]. In particular, all HCWs were subject to mandatory testing for COVID-19 at least on a monthly basis (in case of low occupational biological risk) or weekly (in case of high risk). Moreover, testing was mandatory for COVID-19 contact tracing [17,18].

HCWs are also professionally trained to recognize and characterize clinical symptoms more accurately and reliably than the general population. Therefore, HCWs are an optimal target to assess the impact of pre-existing humoral immunity and mild/asymptomatic SARS-CoV-2 infections against long-COVID-19 syndrome. Estimating long COVID-19 in HCWs is also important to envisage fitness to work and sickness absences among HCWs.

In view of the above, this study aimed to investigate the prevalence of long COVID-19 and associated factors in HCWs of four university hospitals from north-eastern Italy: Trieste, Padua, Verona, and Modena-Reggio Emilia.

## 2. Materials and Methods

The ORCHESTRA multicentric study was approved by the Italian Medicine Agency (AIFA) and the Ethics Committee of Italian National Institute of Infectious Diseases (INMI) Lazzaro Spallanzani. Each center received approval from the local ethical committee.

A survey questionnaire was administered online, between 1 June 2022 and 31 August 2022, to all HCWs employed at the university hospitals of Verona, Padua, Trieste, and Modena-Reggio-Emilia (all located in north-eastern Italy). Informed consent was obtained from all subjects involved in this study.

The survey instrument (Supplementary File) collected information on:

(a) Socio-demographic profile of the responder (including country of birth, ethnicity, marital status, educational level);
(b) Smoking habit;
(c) Any COVID-19 infection, including date of positive swab test, time between 1st and 2nd or 2nd and 3rd infection, days of viral shedding time (VST), eventual hospitalization, length of hospital stay, and admission to intensive care unit;
(d) Date of COVID-19 vaccination, vaccine type, and vaccination status (0, 1, 2, 3, or 4 doses) before any SARS-CoV-2 infection;
(e) Any symptoms developed during acute COVID-19 disease or after first negative swab test;
(f) Post-COVID-19 symptoms persisting after acute disease (i.e., after first negative swab test);
(g) Post-COVID-19 symptoms newly developed after first negative swab;
(h) Long-COVID-19 syndrome (defined as the presence of any symptom) at 30–60 days or 61+ days since after first negative swab;
(i) Any pre-existing condition (defined as morbidities with a medical diagnosis under treatment).

A binary term (yes vs. no) was created to account for the presence of any comorbidities if the responder was undergoing any of the following:

(a) Diabetes therapy;
(b) Cardio-vascular therapy;
(c) Respiratory therapy;
(d) Liver therapy;
(e) Neurological therapy;
(f) Rheumatologic therapy;
(g) Immune-depression (sickle-cell disease, splenectomy, congenital immune-deficiency, HIV, other, none, unanswered)

The date of the positive swab was considered to establish the period of infection, the time since last dose of COVID-19 vaccine received until the next SARS-CoV-2 infection, and the number of primary infections, first reinfection and secondary reinfection. Since swab testing against SARS-CoV-2 only started on 1 March 2020 in Italy, 40 positive swabs for primary SARS-CoV-2 infections erroneously dated between 1 January 2020 and 28 February 2020 were still considered for the descriptive analysis of symptoms, but discarded from any analysis on timeline of infection (COVID-19 waves and time since last COVID-19 vaccine dose received until next infection).

Likewise, the date of any COVID-19 vaccine dose (from 1st to 4th) was used to establish the number of doses received and the timeline since the last dose until next SARS-CoV-2 infection.

### 2.1. Study Endpoints

The presence and duration of any self-reported symptoms following a first negative swab was examined, creating two dichotomized endpoints:

- At 30–60 days: (yes vs. no);
- At 60+ days (yes vs. no).

Any individual symptom after any COVID-19 episode (1st, 2nd or 3rd) was investigated as follows:

- Overall symptoms present after first negative swab for COVID-19 (yes vs. no);
- Symptoms persisting since acute COVID-19 (yes vs. no);
- Symptoms newly developed after first negative swab for COVID-19, but not present during acute disease (yes. vs. no).

Whilst information on long COVID-19 at 30–60 days was available for all HCWs infected at least once (N = 2401) and for 99.7% (N = 2394/2401) at 61+ days, individual acute as well as post-COVID-19 symptoms were not available for 432 HCWs, 121 and 98 of whom still reported long COVID-19 syndrome at 30–60 days and at 61+ days, respectively.

The rates of acute as well as overall post-COVID-19 symptoms were calculated using HCWs that had only been infected with SARS-CoV-2 once or twice as the denominator. The rate of persisting symptoms was calculated using all HCWs reporting symptoms during acute COVID-19 as the denominator. The rate of novel symptoms was calculated using HCWs without symptoms during acute COVID-19 as the denominator.

Categorical variables were contrasted by chi-square test, whereas continuous terms were compared by the Wilcoxon test *p*-value.

After excluding relatively infrequent symptoms (as hemorrhage, skin lesion, and others) the initial 34 individual post-COVID-19 symptoms were reduced to 31 and grouped into six major categories:

- General infectious symptoms (N = 7);
- Otolaryngological (earn–nose–throat; ENT) symptoms (N = 6);
- Pulmonary symptoms (N = 3);
- Gastro-enteric symptoms (N = 4);
- Psychological symptoms (N = 3);
- Neurological symptoms (N = 8).

### 2.2. Statistical Analysis

Univariable logistic analysis was fitted to test the association between each factor and the persistence of any symptom at 30–60 or 61+ days following recovery from COVID-19 (i.e. first negative swab test), reporting an odds ratio (OR) with a 95% confidence interval (95%CI). The effectiveness of COVID-19 vaccination against long COVID-19 was measured 14+ days after one dose and 7+ days after 2+ doses before any SARS-CoV-2 infection.

A backward stepwise approach was carried out to fit the final multivariable logistic regression model, reporting adjusted odds ratio (aOR) with 95%CI on the risk of any symptoms at 30–60 days or 61+ days since first negative swab for primary COVID-19 infection.

A further multiple logistic model was fitted to test the determinants of long COVID-19 at 30–6 or 61 days following the second COVID-19 event, thereby allowing to test the impact of hybrid humoral immunity provided by vaccination as well as previous SARS-CoV-2 natural infection on the risk of long COVID-19 in reinfected HCWs.

In all univariable and multivariable logistic regression models, standard errors allowed for intragroup correlation within each of the four health centers; hence, individual observations were independent across the four centers (clusters), but not necessarily within each center.

Missing values were excluded and complete case analysis was performed.

The analysis was conducted with Stata 16.3 (Stata Corporation, College Station, TX, USA).

## 3. Results

### 3.1. Descriptive Analysis

#### 3.1.1. Socio-Demographic Profile and Medical History of Interviewees

Table 1 shows the profile of the responders (N = 5432) who returned a usable survey tool. As can be seen, the HCW responders were mostly employed in Verona (55.6%), followed by Padua (22.2%), Trieste (18.1%), and Modena-Reggio Emilia (4.6%). The response rates by research center were 15.8% (=983/6230) for Trieste, 36.6% (=2996/8183) for Verona, 17.9% (=1206/6728) for Padua, and 40.5% (=247/610) for Modena Reggio-Emilia.

The HCWs were predominantly female (75.9% = 4122/5432) and had a median age of 47 years (42 years of age for males versus 48 years for females; *p* < 0.001). At the time of the survey, 70.7% of the respondents were vaccinated with three doses, with a lower prevalence among males (66.1%) than females (72.0%) (*p* < 0.001). Among the HCWs, 18.8% were unvaccinated, with a higher proportion of unvaccinated among males (23.9 %) than females (17.2%) (*p* < 0.001).

The majority of responders (55%) were never infected with SARS-CoV-2; 2163 (39.8%) were infected only once; 230 (4.2%) only infected twice; and only 8 HCWs were infected three times. The median time between first and second infection was 411 days, whereas it was 284 days between second and third COVID-19 episodes, with no difference by sex. The majority (46.7%) of responders were married (42.9% males vs. 47.9% females), 31.0% were single (37.2% males vs. 29.9% females), 13.5% lived with someone (15.5% males vs. 12.8% females), and 8.8% were widowed/separated/divorced (4.4% males vs. 10.4% females).

The majority of the interviewees (42.5%) had a university education, with a higher proportion among males (45.2) than females (41.6%). Moreover, 22.7% of the HCWs had a postgraduate education, with a higher prevalence among males (30.5%) than females (20.2%). The predominant ethnicity was Caucasian (88.9%) and 65.2% of the HCWs had a BMI of 18–25 kg/m^2^, with a higher prevalence of BMI = 25–30 among males (33.1%) than females (19.3%).

The prevalence of smokers and ex-smokers was 18.1% and 18.8%, respectively, with slightly higher figures among males (21.0% and 20.1% respectively) than females (17.2% and 18.4% respectively). Current smokers had smoked for a median of 20 years, with a longer amount of time among females (median: 20 years) than males (median: 15 years) (*p* < 0.001). HCWs smoked a median number of 7 cigarettes per day (8.5 for males vs. 7 for females) (*p* = 0.135). Ex-smokers stopped smoking after a median time of 10 years, 8 years for males versus 11 years for females (*p* = 0.062).

Supplementary Table S1 shows the socio-demographic profile of the respondents broken down by center. As can be observed, the distribution of the study population was rather unbalanced not only in terms of the number of respondents, but also in terms of its composition. In particular, 98.2% of HCWs from Modena-Reggio Emilia aged <40 years, since 95.5% among them were postgraduate medical trainees, typically younger. By contrast, undergraduate students were only represented in Verona (7.7%), where the percentage of administrative staff (11.5%) was also slightly higher than Padua or Trieste. The percentage of nurses was instead considerably higher in Padua (48.5%) compared to the other three centers.

#### 3.1.2. COVID-19 Vaccine Uptake

Supplementary Table S2 and Supplementary Figure S1 show COVID-19 vaccine uptake among HCWs. All HCWs were immunized with m-RNA vaccines, predominantly with Comirnaty (Pfizer BioNTech) and marginally by Spikevax (Moderna). The national vaccination campaign was in fact started on 27 December 2021 in Italy and at the beginning of the Omicron transmission period (15 December 2021), about 60% of HCWs were immunized with three doses, a proportion that increased up to 70% by the end of the study period (31 August 2022).

#### 3.1.3. SARS-CoV-2 Infections

As can be seen from Table 2, 2163 HCWs were only infected with SARS-CoV-2 once, 230 were only infected twice, and 8 were infected three times. Among the HCWs infected only once, 75% were immunized with the booster before their first COVID-19 infection, against 51.3% among HCWs that were infected only twice. Moreover, the two most transmissible COVID-19 waves were 1 November 2020–31 May 2021 (broadly corresponding to the Alpha transmission period), when 16.7% of primary infections and 6.0% of reinfections occurred, and from 1 December 2021 onward (Omicron transmission period), accounting for 70.6% of primary SARS-CoV-2 infections and 91.5% of reinfections. By 1 January 2021, at the start of the COVID-19 vaccination campaign, 9.3% HCWs (=507/5432) had already been infected with SARS-CoV-2 once in the present study.

Table 2 also shows that the time between last COVID-19 vaccine dose and infection progressively decreased with higher number of doses of vaccines received, both for primary infections and reinfections. The median VST progressively diminished in HCWs that were infected with SARS-CoV-2 only once (11 days; IQR: 9; 16); two (8 days; IQR: 7; 12); or three times (7 days; IQR: 7: 10.5). Total COVID-19-related hospitalizations were 21 among HCWs infected with SARS-CoV-2 only once against one in those infected only twice and zero among those infected three times (Table 2). The median length of hospital stay following COVID-19-related admission was 10 (IQR: 5; 15) days in those infected only once (Table 2).

**Figure 1:**
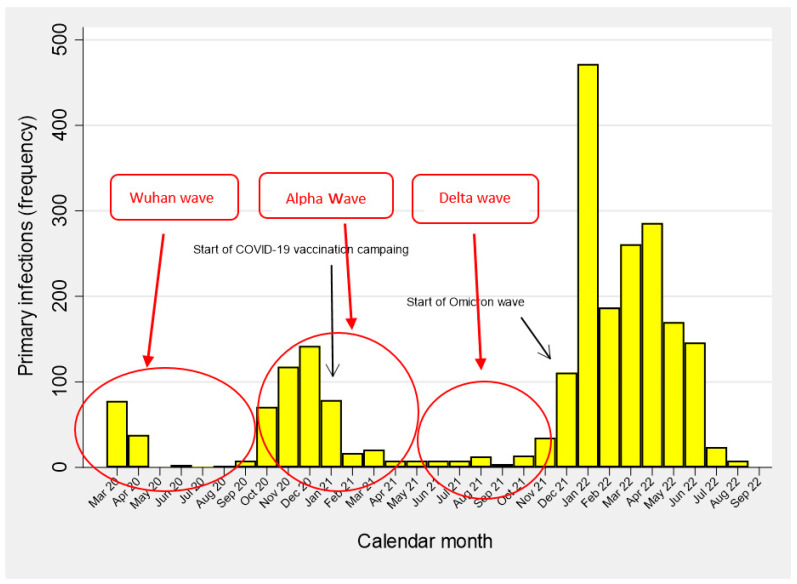
Frequency distributions of primary SARS-CoV-2 infections (N = 2401) over time (March 2020–September 2022). Healthcare workers from all 4 health centers combined (N = 5432).

**Figure 2:**
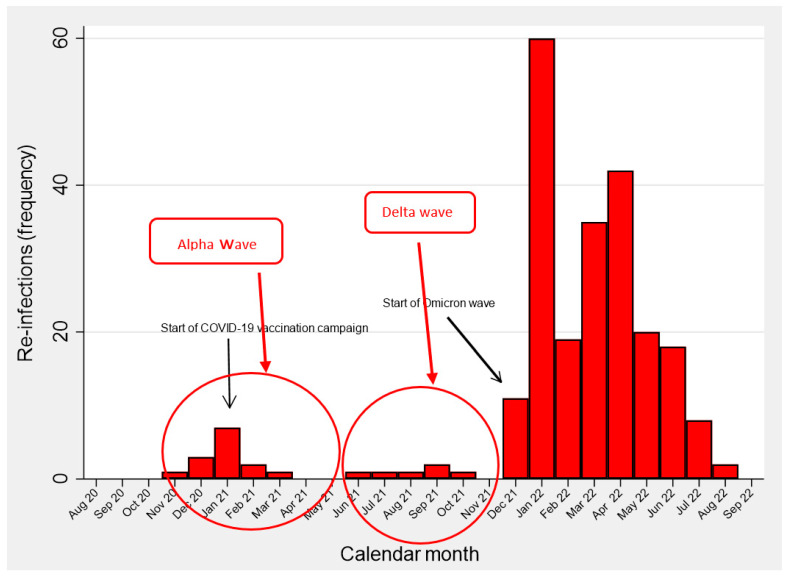
Frequency distributions of SARS-CoV-2 re-infections (N = 238) over time (March 2020–September 2022). Healthcare workers from all 4 health centers combined (N = 5432).

Figure 1 shows the epidemic curve for primary COVID-19 infections over time, with the temporal distribution of waves broadly corresponding to the transmission periods of the main SARS-CoV-2 variants (wild-type, Alpha, Delta, Omicron). As can be noted, the Alpha (October 2020–May 2021) but especially the Omicron transmission periods, accounted for the vast majority of primary infections. Likewise, the transmission period of the two above variants corresponded to major waves of reinfections, particularly since December 2021 (Figure 2).

As can be seen from Table 3, the highest rate of COVID-19 hospitalization was observed during the Wuhan transmission period (4.6% = 10/217), followed by the Delta (2.5% = 2/81), Alpha (0.5% = 2/389), and Omicron (0.4% = 6/1644) waves, in decreasing order.

#### 3.1.4. Acute and Post-COVID-19 Symptoms

The most frequent post-COVID-19 symptom was asthenia (30.3%), followed by myalgia (13.7%), cough (12.4%), dyspnea (10.2%), concentration deficit (8.1%), headache (7.3%), and anosmia (6.5%) in decreasing order (Table 4). Asthenia (14.2%), cough (8.9%), myalgia (3.0%), dyspnea (2.0%), anosmia (1.8%), concentration deficit (1.5%), and headache (1.4%) were the most frequent single-symptoms self-reported by HCWs. As can be noted from Table 4, when two symptoms were coexistent, asthenia was almost consistently present, in combination with either myalgia (6.0%), cough (4.7%), dyspnea (3.8%), headache (2.6%), or concentration deficit (2.4%). In the case of three symptoms, asthenia was always included, in combination with either myalgia and dyspnea (1.6%), myalgia and cough (1.2%), anosmia and dysgeusia (1.1%), myalgia and concentration deficit (1.1%), and myalgia and headache (1.1%) (Table 4).

Supplementary Figure S2 shows the distribution of symptoms during acute COVID-19, and first versus second infection. As can be noted, for both primary infection as well as reinfection, the most prevalent symptoms were those typical of respiratory diseases, i.e., asthenia, fever, myalgia, cough, headache, shivering, rhinorrhea, rhinitis, dysgeusia, and anosmia, in decreasing order of prevalence. It can be noted that the proportion of symptoms was consistently lower upon COVID-19 reinfection, particularly for dysgeusia and anosmia.

Supplementary Figure S3 shows the rates of individual post-COVID-19 symptoms (persisting or novel combined) following the first COVID-19 episode, also broken down by long COVID-19 at 30–60 days or 61+ days. It can be seen how the most prevalent post-COVID-19 symptom was asthenia, followed by myalgia, cough, dyspnea, concentration deficit, headache, anosmia, and dysgeusia in decreasing order of prevalence. The prevalence of post-COVID-19 symptoms at 30–60 days consistently reduced at 61+ days.

**Figure 3:**
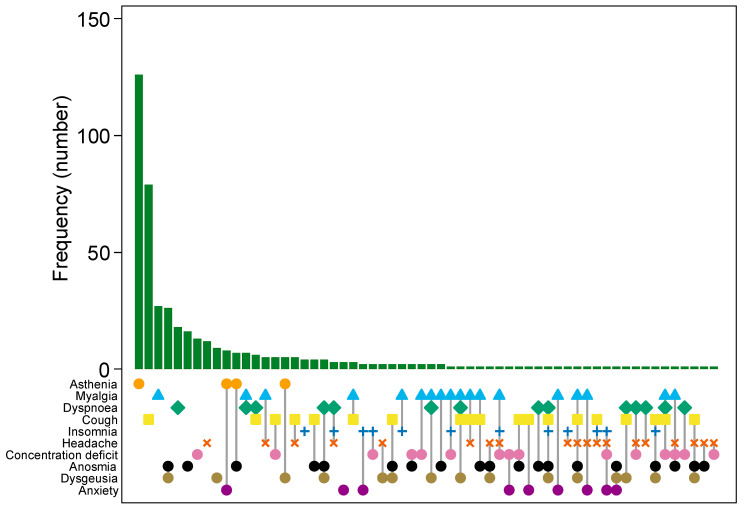
UpSetPlot displaying the 60 most frequently occurring subsets of post-COVID-19 symptoms. Each symptom is represented by a specific coloured shape. 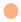 = Asthenia; 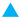 = Myalgia; 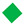 = Dyspnoea; 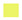 = Cough; 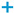 = Insomnia; 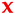 = Headache; 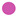 = Concentration deficit; 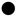 = Anosmia; 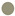 = Dysgeusia; 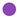 = Anxiety.

Figure 3 shows an UpSetPlot with the most frequent subsets of post-COVID-19 symptoms overall (persisting or novel).

As can also be appreciated from above Table 4, the most frequent single post-COVID-19 symptoms were asthenia, cough, myalgia, dyspnea, anosmia, concentration deficit, and headache, in decreasing order of prevalence. Asthenia was also the most frequent post-COVID-19 condition in association with another symptom (myalgia, cough, dyspnea, headache, and concentration deficit) and was consistently included when three of the most commonly reported symptoms were combined, with myalgia being the second most common sequela with three symptom combinations. As can be seen from Table 4, asthenia, myalgia, dyspnea, and cough were the most frequently reported post-COVID-19 symptoms in total, as single symptoms or in combination with other symptoms.

Table 5 shows the Spearman correlation of the most frequent symptoms following a first negative swab after SARS-CoV-2 infection (first or second). It can be noted that, following the first COVID-19 event, the correlation coefficient was higher for asthenia/myalgia (Rho = 0.412), asthenia/dyspnea (Rho = 0.328), asthenia/concentration deficit (Rho = 0.321), insomnia/concentration deficit (Rho = 0.276), dyspnea/myalgia (Rho = 0.247), insomnia/asthenia (Rho = 0.241), asthenia/headache (Rho = 0.238), headache/insomnia (Rho = 0.225), and myalgia/headache (Rho = 0.225). Stronger significantly correlations after a second COVID-19 episode were found for asthenia/dysgeusia (Rho = 0.268), asthenia/cough (Rho = 0.247), concentration deficit/anosmia (Rho = 0.238), followed by dyspnea/anosmia (Rho = 0.204), and asthenia/headache (Rho = 0.186) (Table 5).

**Figure 4:**
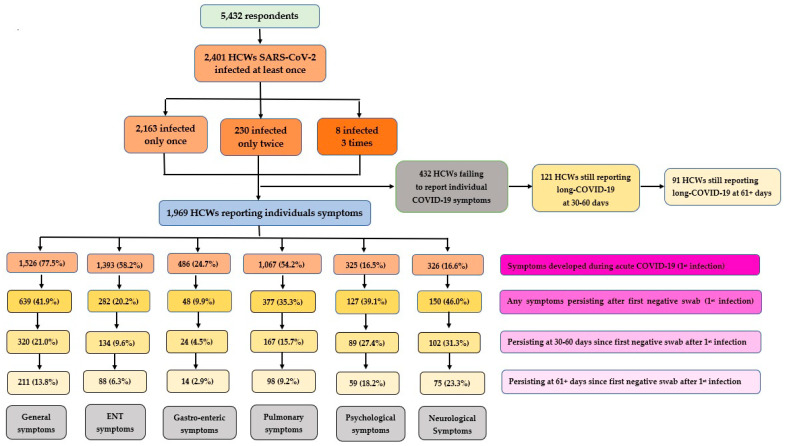
Number and percentage of symptoms reported during acute COVID-19 and subsequently persisting after first negative swab test, among 2401 healthcare workers infected with SARS-CoV-2 at least once. Individual symptoms not available (missing) for 432 healthcare workers.

Figure 4 shows the frequency of symptoms persisting (all; at 30–60 days; at 61+ days) since a first negative swab following a primary COVID-19 episode, grouped by six major categories: general; pulmonary; otolaryngological; gastro-enteric; psychological; and neurological. It can be appreciated that, although less prevalent during acute disease, neurological and psychological symptoms were more likely to persist over time, with rates of 31.3% vs. 27.4% at 30–60 days and 23.3% vs. 18.2% at 61+ days, respectively. The proportion of general symptoms persisting after a first negative swab was 21.0% at 30–60 days and 13.8% at 61+ days.

**Figure 5:**
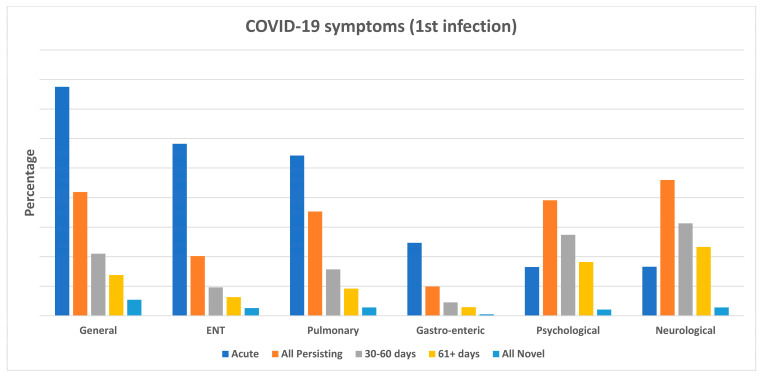
Prevalence of symptom groups, by acute, overall persisting, persisting at 30–60 days, at 61+ days, or as newly developed (regardless of the timeline) following a first negative swab for a primary COVID-19 episode.

Figure 5 shows the prevalence of symptom groups, as acute, overall persisting, and persisting at 30–60 days, at 61+ days, or as newly developed (regardless of the timeline) following a first negative swab for a primary COVID-19 episode. It can be noted once again that, despite being less frequent during the acute disease, the rates of psychological and neurological persisting symptoms increased remarkably at 30–60 and 61+ days since a first negative swab test.

Table 6 shows frequency and proportion of symptom groups: acute; overall post-COVID-19 (persisting or novel) overall; present at 30–60 days; at 61+ days; symptoms persisting since acute disease and the rate of novel symptoms. As already mentioned in Section 2, the rates of acute as well as overall post-COVID-19 symptoms were estimated out of all HCWs infected with SARS-CoV-2 only once or twice. While the rate of persisting symptoms was estimated out of all HCWs reporting symptoms during acute COVID-19, the rate of novel symptoms was estimated out of the HCWs not reporting symptoms during acute disease. As can be seen from Table 6, whilst general (35.1%), ENT (16.2%), or pulmonary (21.0%) symptoms (persisting or novel) were the most commonly reported following a first COVID-19 event, higher rates of neurological and psychological symptoms (persisting or novel) were still present at 30–60 days (67.6% vs. 68.9%, respectively) as well as 61+ days (47.4 vs. 45.5%, respectively). Moreover, higher rates of neurological symptoms (46.0%) were persisting since acute disease, followed by general (41.9%), psychological (39.1%), pulmonary (35.3%), otolaryngological (20.2%), and gastro-enteric (10.5%) symptoms. The rates of novel symptoms were higher for general (17.8%) or otolaryngological (8.9%) symptoms, followed by pulmonary (4.9%) and neurological (4.9%) (Table 6).

Detailed distribution of individual acute and post-COVID-19 symptoms (overall, persisting, novel) at 30–60 days or 61+ days since a first negative swab after primary COVID-19 infection can be seen in Supplementary Table S3. As can be noted, the prevalence of long COVID-19 was 24.0% (=576/2401) during the period of 30–60 days since a first negative swab for a primary COVID-19 event, reducing to 16.2% (=389/2401) at 61+ days, for a 32.5% (=187/576) symptom resolution after 2 months. By contrast, the prevalence was 10.5% (=25/238) during the period of 30–60 days after first negative swab following second infection, further diminishing to 5.4% (=13/238) at 61+ days, for a 48% (=12/25) symptoms resolution after 2 months.

### 3.2. Logistic Regression Analysis

Table 7 shows the prevalence and risk of long COVID-19 at 30–60 or 61+ days since first negative swab test following a primary COVID-19 infection, by explanatory factors.

Table 8 shows the results of the two respective multivariable logistic regression models investigating long COVID-19 at 30–60 or 61+ days since a first negative swab after a primary COVID-19 infection. The risk of long COVID-19 at 30–60 days was significantly higher in HCWs previously hospitalized for COVID-19 (aOR = 3.34; 95%CI: 1.62; 6.89) and in those infected during the early stages of the pandemic, namely during the original Wuhan (aOR = 2.16; 95%CI: 1.14; 4.09) or Alpha (aOR = 2.05; 95%CI: 1.25; 3.38) transmission periods, and progressively increased with VST, especially for 15+ days (aOR = 3.20; 2.07; 4.94). Furthermore, the risk of long COVID-19 at 30–60 days was slightly higher in females (aOR = 1.91; 95%CI: 1.30; 2.80) and in HCWs aged > 40, those with an abnormal BMI, or HCWs that were divorced/separated/cohabitant (aOR = 1.22; 95%CI: 1.15; 1.30). In contrast, long COVID-19 at 30–60 days was less likely in medical trainees (aOR = 0.74; 95%CI: 0.59; 0.93) or undergraduate medical students (aOR = 0.27; 95%CI: 0.19; 0.40). Moreover, HCWs employed in medical (aOR = 0.39; 95%CI: 0.24; 0.62) or surgical wards (aOR = 0.52; 95%CI: 0.36; 0.77) or other HCWs (aOR = 0.64; 95%CI: 0.52; 0.78) were less likely to experience any long COVID-19 symptoms at 30–60 days than those employed in administrative services.

Table 8 also shows risk factors for any long COVID-19 symptom at 61+ days since a first negative swab after a primary COVID-19 infection. Whilst the effect of early pandemic waves lost statistical significance, the effect size of previous hospitalization for COVID-19 (aOR = 4.09; 95%CI: 2.25; 7.44) increased with the VST—especially 15+ days (aOR = 5.00; 95%CI: 2.84; 8.81)—and female sex (aOR = 2.14; 95%CI: 1.69; 2.71). Age> 40 and BMI >30 were confirmed to be significant risk factors for long COVID-19 at 61+ days, along with non-Caucasian ethnicity (aOR = 1.83; 95%CI: 1.41; 2.36). Furthermore, HCWs vaccinated with two doses (aOR = 0.60; 95%CI: 0.36; 0.99) and postgraduate medical trainees (aOR = 0.47; 95%CI: 0.29; 0.77) were less likely to experience long-COVID-19 syndrome at 61+ days, along with other clinical professionals such as laboratorists (aOR = 0.37; 95%CI: 0.21; 0.67), nurse aids (aOR = 0.53; 95%CI: 0.38; 0.72), midwives (aOR = 0.37; 95%CI: 0.21; 0.64), or health technicians (aOR = 0.73; 95%CI: 0.59; 0.91).

Table 9 shows the frequency distribution and univariable as well as multivariable logistic regression analysis of the risk of long COVID-19 at 30–60 days and 61+ days following a second COVID-19 infection. As can be seen, the risk of long COVID-19 increased consistently for a VST of 15+ days at 30–60 days (aOR = 4.76; 95%CI: 2.81; 8.07) or for BMI 26–30 (aOR = 4.66; 95%CI: 3.54; 6.14) at 61+ days. Albeit <1 for any dose of COVID-19 vaccine, the OR for long COVID-19 at 30–60 days as well as 61+ days never reached statistical significance in either the univariable or multivariable analysis (Table 9).

## 4. Discussion

### 4.1. Key Findings

In the present study, 2401 HCWs were infected with SARS-CoV-2 at least once; 238 were infected at least twice; and 8 were infected three times. The prevalence of long COVID-19 was 24.0% at 30–60 days since the end of a primary SARS-CoV-2 infection versus 16.3% at 61+ days, for an average resolution of 32.5% of symptoms after 2 months. The prevalence of long COVID-19 was much lower (10.5%) at 30–60 days after a second SARS-CoV-2 infection, further reducing to 5.5% at 61+ days, for an average symptom resolution of 48% at 2 months since a first negative swab test result.

The most frequent post-COVID-19 symptom was asthenia, followed by myalgia, cough, dyspnea, concentration deficit, headache, and anosmia, in decreasing order of prevalence. Asthenia, cough, myalgia, dyspnea, anosmia, concentration deficit, and headache were the most frequent singleton symptoms self-reported by HCWs in the present study. When two symptoms were coexisting, asthenia was almost constantly present, in combination with either myalgia, cough, dyspnea, headache, or concentration deficit. In cases with three symptoms coexisting, asthenia was always included, in combination with either myalgia and dyspnea, myalgia and cough, anosmia and dysgeusia, myalgia and concentration deficit, or myalgia and headache.

Whilst general, otolaryngological, or pulmonary symptoms (persisting or novel) were most commonly reported following a first COVID-19 event, higher rates of neurological and psychological symptoms (persisting or novel) were still present at 30–60 days (67.6% vs. 68.9%, respectively) as well as 61+ days (47.4 vs. 45.5%, respectively) after first negative swab. Moreover, higher rates of neurological symptoms were persisting since acute COVID-19, followed by general, psychological, pulmonary, otolaryngological, and gastro-enteric symptoms, in decreasing order of prevalence.

The main determinants of long-COVID-19 syndrome at 30–60 as well as 61+ days were COVID-19-associated hospitalization, 15+ days VST, female sex, age > 40 years, abnormal BMI, or occupation as administrative staff (reference category). HCWs infected with SARS-CoV-2 during the early pandemic waves (in the pre-vaccination era or early phases of the vaccination campaign) were more likely to experience a long-COVID-19 syndrome at 30–60 days, but not at 61+ days. In contrast, the risk of long COVID-19—both at 30–60 as well as 61+ days since first negative swab—was consistently lower for medical students and postgraduate medical trainees.

### 4.2. Prevalence of Long COVID-19

Contrasting the prevalence of long COVID-19 is complicated by several major hurdles, namely definition employed for long COVID; different criteria for the starting of the follow-up time, i.e., either disease diagnosis, symptom onset, or end of acute disease (communicability window); different time lags for symptom assessment; various study populations; variable COVID-19 vaccine uptakes; different geographical areas; and study period (reflecting different SARS-CoV-2 variants involved), among others. Furthermore, the assessment of long COVID-19 is inherently affected by bias, since it relies on self-reported symptoms.

A systematic review of 120 studies published between 1 January 2020 and 2 November 2021 (pre-Omicron period) reported a very wide pooled prevalence of long COVID-19 ranging from 0 to 93%, with estimates from routine healthcare records (13.6%) typically lower than those relying on self-reported symptoms (43.9%) [10].

Another systematic review of 194 studies published before January 2022, including 735,006 patients in total, estimated a 45% proportion of COVID-19 survivors experiencing at least one persisting symptom after a mean follow-up time of 126 days (range from 28+ to 387 days) [13]. The discrepancy between in the latter estimates and the prevalence of long COVID-19 in the present study is likely explained by different study populations. The above systematic reviews only covered the pre-omicron transmission periods and mainly or predominantly assessed long COVID-19 in the general population (excluding HCWs); furthermore, it also included Asian populations, where in some areas the rates of severe COVID-19 were particularly high during the Delta wave [10,13,19]. In contrast, the present survey was conducted on HCWs, whose respective vaccination uptake is typically higher than that the general population. HCWs are also part of the active workforce, and hence featured by “healthy worker” effect. For instance, in a study of 679 HCWs from India conducted between July and October 2021, the overall prevalence of long-COVID sequelae was 30.3%—and hence closer to the present survey [4].

It was estimated that 34.5% patients do not recover a state of full health/fitness at 12+ weeks since acute COVID-19 infection according to the above systematic review on 120 studies on the general population published in the pre-Omicron period [10]. In the present study, 62.5% HCWs were still experiencing some symptoms 2 months since a first negative swab after 1st SARS-CoV-2 infection, a rate which may have further reduced at 3 months. In fact, in the above study from Trieste, 75% of outpatients reporting any symptoms after a median time of 49 days since COVID-19 diagnosis recovered a status of full health at second follow up at 15 months [16].

### 4.3. Post-COVID-19 Symptoms

The assessment of post-COVID-19 symptoms is rather variable in the open literature, since symptoms persisting or newly developed after the end of the communicability window are seldom distinguished. However, asthenia (21.6%), dyspnea (14.9%), insomnia (13.2%), and arthralgia/myalgia (10.6%) were the most common symptoms estimated by the above systematic review including 120 studies of the general population [10]. Asthenia, insomnia and dyspnea are the most prevalent post-COVID-19 symptoms both among hospitalized, non-hospitalized, and mixed cohorts according to another systematic review [13].

Asthenia, myalgia, cough, and dyspnea were also the most frequently overall symptoms (persisting or novel) following the first COVID-19 episode in the present survey. Moreover, the most common individual symptoms persisting at 30–60 days since acute disease in the present study were asthenia, myalgia, dyspnea, concentration deficit, headache, dysgeusia, cough, insomnia, and anxiety, in decreasing order of prevalence. Likewise, in the above study on 679 HCWs from India, the most common symptom was asthenia (11.5%), followed by insomnia (8.5%), exercise dyspnea (6%), and arthralgia (5%) [4].

Whilst cough and dyspnea can be objectively assessed, asthenia, insomnia, myalgia, and concentration deficit are subjective symptoms potentially affected by self-reporting bias.

Asthenia (or fatigue), a common sickness reaction to various pathogens, is considered a normal homeostatic alert which is functional to energy preservation during acute infections, normally in combination with other typical symptoms (myalgia, dyspnea, insomnia, and cognitive deficits, among others) [20,21]. Some general symptoms can persist for weeks or months following acute infectious diseases. A meta-analysis of studies reported an overall prevalence of asthenia during acute COVID-19 of 23% (95%CI: 15–33%) [22].

Post-COVID-19 asthenia—sharing several clinical patterns with myalgic encephalomyelitis/chronic fatigue syndrome—can have multiple putative factors—including the hypo-metabolism of the frontal lobe and cerebellum, among others [14,16]. Post-COVID-19 asthenia is inadequately assessed, often by telephone interviews, mobile apps, medical records, or standard questionnaires [21].

Asthenia, accounting for a third access to primary care services, can be sustained by either physical or psychiatric conditions (mainly depression) [21,22,23,24]. Pre-existing depression was in fact a significant risk factor for long-COVID-19 syndrome at 15 months and psychiatric symptoms at 49 days since COVID-19 diagnosis in the above outpatient study from the general population of Trieste (north-eastern Italy) [16].

In absence of alternative explanations, and in coexistence with other typical general symptoms, a post-infective fatigue syndrome could be considered following COVID-19 [21,25,26]. Sandler et al. proposed some criteria to apply post-infective COVID-19 asthenia [21]. However, the latter criteria failed to consider symptoms newly developed after acute COVID-19 and relied on an old definition of post-infective fatigue, considering a 6-month follow-up time [26]. Post-COVID-19 asthenia following the re-adaptation of the above criteria by Sandler et al. could be suspected if asthenia fulfills the first three in addition to either of the last two conditions [21]:

- Is a chronic and dominant symptom;
- Is frequently combined with other symptoms;
- Impacts the majority of everyday life activities;
- Developed during acute COVID-19, persisting at least 30 days afterwards;
- Did not manifest during acute COVID-19, but newly developed shortly after the first negative swab, persisting at least 30 days afterwards.

The assessment of asthenia should take into account also predisposing, precipitating, and perpetuating factors [21]. COVID-19 can act as a precipitating factor (catalyzer) for pre-existing and predisposing conditions (genetics, co-morbidities, or psycho-social states) [27,28], with or without coexistent triggering life tragedies (e.g., mourning, life failures, job dismissal, etc.) [29]. A number of conditions subsequently developing as insomnia [30], dysautonomia [31], endocrine disruption [32], or mood disorders [33] can then act as factors perpetuating post-COVID-19 asthenia [21].

Long COVID-19 displays changes in pulmonary patterns that are similar to those of respiratory diseases sustained by other coronaviruses (SARS-CoV-1 and MERS-CoV) [13,34], with typical endothelial damage and lung inflammation caused by viral replication and a cytokine storm potentially evolving into fibrotic scars, which may account for long-term respiratory symptoms as cough and dyspnea. However, most patients suffering from post-COVID-19 dyspnea do not exhibit the radiological signs of pulmonary damage [14,35,36]. Pathology was reported only by a small number of studies, with lung pathology being the most commonly reported (38.9%), followed by heart (6.0%), or neurological pathology (5.3%) [10].

Cognitive post-COVID-19 symptoms such as concentration deficit, memory loss, or headache may be sequelae of direct viral encephalitis, systemic inflammation, or cerebrovascular alterations inflicted by the respective severe acute disease [14].

Semi-structured diagnostic tools, such as the Structured Clinical Interview for Neurasthenia (SCIN) [37] and the Composite International Diagnostic Interview (CIDI) are available to screen potential post-COVID-19 symptoms, disentangling asthenia sustained by fatigue states from psychiatric disorders for research purposes [20]. Likewise, other major post-COVID-19 symptoms such as myalgia/arthralgia, insomnia, and concentration deficit could be evaluated by validated screening tools, including the Structured Diagnostic Interview for Sleep Patterns and Disorders (DISP) or the Composite International Diagnostic Instrument (CIDI) [21].

Following the identification of a potential post-COVID-19 syndrome, further clinical (respiratory, cardiac, or neurological) investigations may be considered, along with laboratory testing and radiological imaging.

An online survey conducted during the first year of the pandemic on 3762 COVID-19 survivors from 56 countries, with confirmed or suspected SARS-Cov-2 infection before June 2020, with an illness enduring >28 days, reported that >91% respondents still had symptoms persisting 7 months after diagnosis [38]. However, as mentioned above, the risk of severe COVID-19 with Omicron is currently very low and most patients who developed the syndrome following SARS-CoV-2 infection in the early pandemic waves tended to return to a state of full health with time [13,16]. A cost-effective approach to screen long COVID-19 during the Omicron period may be restricted to vulnerable patients (immuno-compromised, elderly, and/or patients with co-morbidities) developing severe disease or exhibiting prolonged VST.

### 4.4. Risk Factors for Long COVID-19

The risk of long COVID increased with the severity of acute disease in the present study. The hospitalization and severity of acute infection were key determinants of long COVID-19 according to the above systematic review on 120 studies [13], and the risk further increased with admission to intensive care unit (ICU) or mechanical ventilation [4,14,15]. Severe COVID-19, which is more likely to develop in vulnerable patients, including elderly, immunocompromised, and/or patients with pre-existing cardio-respiratory conditions, can heal with fibrotic lung scarring, compromising lung capacity and determining chronic post-COVID-19 sequelae as dyspnea, cough, and asthenia [9,35].

HCWs infected during the early pandemic waves (Wuhan and Alpha transmission periods) were more likely to experience long COVID-19 at 30–60 days in the present survey. The different SARS-CoV-2 variants that emerged over the course of the pandemic exhibited different levels of transmissibility and virulence, impacting both acute disease and post-COVID-19 sequelae [14]. Whilst during the Omicron transmission period there is evidence of a reduced risk of both hospitalization and post-COVID-19 symptoms [12,15,18,39], early variants were associated with higher disease severity [13,38,40,41]. According to a systematic review of 42 studies including 6174,807 patients, Omicron had a 56% lower hospitalization rate, 54% lower risk of ICU admission, and 61% lower mortality rate than the Delta variant [41]. Moreover, in a UK case–control study using self-reported data from the COVID Symptom Study app of King’s College London, 56,003 adults infected with SARS-CoV-2 between 20 December 2021 and 9 March 2022—when more than 70% of UK cases were caused by Omicron—were contrasted with 41,361 UK adult cases infected between 1 June 2021 and 27 November 2021—when more than 70% of cases were attributable to the Delta variant. The risk of long-COVID-19 syndrome was significantly lower with Omicron compared to Delta for any vaccine timing (<3 months vs. 3–6 months vs. >6 months since immunization) and age (18–59 vs. 60+ years) of patients in the latter UK study [12].

However, the effect of the pandemic wave was not confirmed at 61+ days in the present investigation, suggesting the prevailing effect of disease severity over the timeline of SARS-CoV-2 infection.

In the present study, the vaccination status with two doses reduced the risk of long COVID-19 at both 30–60 as well as 61+ days following a first SARS-CoV-2 infection. The national vaccination campaign started on 27 December 2020 in Italy, and by 1 March 2021, in the middle of Alpha wave, more than 50% of HCWs already received a second dose of COVID-19 vaccine; hence, this likely reduced the risk of severe disease in those immunized. Conversely, the booster dose was started by October 2021 onward, near the end of Delta wave, and just before the start of the Omicron transmission period, when the risk of severe disease decreased substantially.

However, the prevalence of long COVID-19 after a second SARS-CoV-2 infection was remarkably lower regardless of vaccination status, suggesting that hybrid immunity did not provide further protection as compared to immunity mounted by either natural infection or vaccination.

Prolonged (15+ days) VST was a major determinant of long COVID-19 at 30–60 days as well as 61+ days in the present study, both following first and second SARS-CoV-2 infection. Longer VST may increase the inflammatory reaction against viral replication, thereby enhancing the risk of long COVID-19. Moreover, a higher viral load during acute disease may reflect patient vulnerability to SARS-CoV-2 infection and an increased risk of progressing to severe COVID-19 [42]. Likewise, VST reflects humoral immunity, as it was found to be inversely associated with an increasing number of doses of COVID-19 vaccine in high-risk patients [39]—although the latter finding was not confirmed for low-risk patients affected by mild–moderate COVID-19 in a recent clinical trial [43].

Within our cohort, age > 40 and abnormal BMI increased the risk of long COVID-19 after first infection and a BMI of 26–30 increased the risk at 61+ days, also following second SARS-CoV-2 infection. BMI, advanced age, and co-morbidities are established risk factors for both SARS-CoV-2 infection and severe disease, also among the vaccinated workforce [44]. Likewise, in the present study the risk of long COVID-19 was lower in postgraduate medical trainees or undergraduate medical students, who are typically younger.

Finally, females were more likely to report long COVID-19 in the present study. This is in line with the open literature, consistently showing that the risk of long COVID-19 is higher in females [4,10,15,45,46,47]. Although hormone-dependent mechanistic hypotheses have been proposed, sex-dependent self-reporting bias shall not been ruled out either, considering the fact that sex-dependent discrepancy in medical care already existed long before the pandemic [15,16]. Not to mention that, whilst females were more likely to report long COVID-19, males were more likely to develop severe disease during the pre-Omicron period due to multiple risk factors including androgen-driven pathogenesis [14,44,48]. A similar self-reporting biases may also at least partially explain the consistently higher risk of long COVID-19 reported by administrative staff in the present survey.

### 4.5. Strengths and Limitations

This study investigated the impact of long-COVID-19 syndrome in a relatively large sample of HCWs, with a detailed collection of symptoms and risk estimates adjusted for a high number of potential confounders.

However, this investigation was restricted to the sub-sample of responders, which may not necessarily be representative of the entire healthcare workforce of the four health centers. Furthermore, the response rate was unbalanced across centers, with different units unevenly contributing to the final sample.

As already mentioned, post-COVID-19 symptoms are subject to potential self-reporting bias. Recall bias may also be involved in other information collected by the questionnaire, such as VST, which was provided by the patients themselves, as information on the date of the first negative swab test was not available.

We estimated SARS-CoV-2 infections and COVID-19 vaccine uptake based on the date of infections and date of vaccination. This could again be subject to potential recall bias, as missing information on latter dates may not necessarily signify zero infections or zero vaccine doses. The number of unvaccinated and number of HCWs uninfected may therefore be slightly inflated. Moreover, information on the antibody level before any SARS-CoV-2 infection was not available.

The risk of long COVID-19 following a second SARS-CoV-2 infection was estimated on a multiple logistic model fitted onto just 214 complete observations at 30–60 days versus 141 complete observations at 61+ days. An insufficient statistical power of the latter models may account for the lack of statistical significance for some risk estimates.

Finally, in the present study, multivariable regression logistic regression models were built up by backward stepwise selection, an automated procedure failing to consider the role of potential mediators in the causal pathway between relevant risk factors and long-COVID-19 syndrome. Although advanced statistical methods are welcome in future research to confirm the causality of long COVID-19, the main risk factors identified by the present study seem plausible.

## 5. Conclusions

The prevalence of long COVID-19 was 24.0% at 30–60 days after the end of communicability window of a primary SARS-CoV-2 infection, reducing to 16.3% at 61+ days, for an average resolution of symptoms of 32.5% of at 2 months. Conversely, the prevalence of long COVID-19 was much lower (10.5%) at 30–60 days since a first negative swab following the second SARS-CoV-2 infection, further reducing to 5.5% at 61+ days, for a 48% rate symptom resolution at 2 months.

The most frequent symptom after first COVID-19 event was asthenia, followed by myalgia, cough, dyspnea, concentration deficit, headache, and anosmia, in decreasing order of prevalence.

The risk of long COVID-19 at 30–60 days increased particularly in HCWs hospitalized for COVID-19, infected with SARS-CoV-2 during the early pandemic waves, and those with a longer VST.

The risk of long COVID-19 seems inversely influenced by the humoral immunity level, mounted either by SARS-CoV-2 natural infection of vaccination. However, the prevalence of long COVID-19 after second SARS-CoV-2 infection was remarkably lower regardless of the vaccination status, suggesting that hybrid immunity did not provide further protection compared to immunity mounted by either natural infection or vaccination separately.

Since the risk of long COVID-19 is currently low with Omicron and patients who developed the syndrome following SARS-CoV-2 infection in the early stages of the pandemic tend to return to a state of full health with time, a cost-effective approach to screen post-COVID-19 symptoms could be restricted to vulnerable patients developing severe COVID-19 and/or with prolonged VST.5.

## Figures and Tables

**Table 1:** Distribution of variables by sex of respondents. Number (N); column percentage (%); mean ± standard deviation (SD); and median with interquartile rage (IQR). Chi-square (for categorical terms) or Wilcoxon test *p*-value (for continuous terms). M = missing data.

Terms	Strata		Total (Tot = 5432) N (%)	Males (Tot = 1310) N (%)	Females (Tot = 4122) N (%)	*p*-Value
**Sex**	**Males**		1310 (24.1)			
	**Females**		4122 (75.9)			
**Health ** **center**	**Verona**		2996 (55.1)	716 (54.7)	2280 (55.3)	<0.001
	**Padua**		1206 (22.2)	227 (17.3)	979 (23.7)	
	**Trieste**		983 (18.1)	271 (20.7)	712 (17.3)	
	**Modena-Reggio Emilia**		247 (4.6)	96 (7.3)	151 (3.7)	
**Age** (years) (M: 830)	**Mean ± SD**		44.6 ± 12.0	43.2 ± 12.8	45.1 ± 11.7	
	**Median (IQR)**		47 (33; 55)	42 (31; 55)	48 (34; 55)	<0.001
	**<40**		1716 (37.3)	511 (46.6)	1205 (34.4)	<0.001
	**40–54**		1655 (36.0)	305 (27.8)	1350 (38.5)	
	**55+**		1231 (26.7)	281 (25.6)	950 (27.1)	
**Doses of** **COVID-19 vaccine** (number)	**0**		1020 (18.8)	313 (23.9)	707 (17.2)	<0.001
	**1**		208 (3.8)	56 (4.3)	152 (3.1)	
	**2**		352 (6.5)	65 (5.0)	287 (7.0)	
	**3**		3839 (70.7)	872 (66.2)	2967 (72.0)	
	**4**		13 (0.2)	4 (0.3)	9 (0.2)	
**COVID-19** **Infections** (number)	**0**		3.058 (56.3)	783 (59.8)	2275 (55.2)	0.027
	**1**		2163 (39.8)	479 (36.6)	1657 (40.2)	
	**2**		230 (4.2)	46 (3.5)	184 (4.5)	
	**3**		8 (0.2)	2 (0.1)	6 (0.1)	
**Time between** **COVID-19 infections** (days)	**1st–2nd** (missing: 33)	**M ± SD**	387.7 ± 225.4	442.4 ± 248.8	373.0 ± 217.1	
		**M (IQR)**	411 (176; 540)	462 (216; 665)	400 (25; 75)	0.042
	**2nd–3rd** (missing: 3)	**M ± SD**	312 ± 183.5	366 ± 251.7	276.3 ± 175.6	
		**M (IQR)**	284 (142.5; 496)	366	284	0.563
**Marital status** (M: 37)	**Single**		1636 (31.0)	473 (37.2)	1163 (29.9)	<0.001
	**Married**		2466 (46.7)	545 (42.9)	1921 (47.9)	
	**Cohabitant**		711 (13.5)	197 (15.5)	514 (12.8)	
	**Divorced/separated/widow**		469 (8.8)	57 (4.4)	412 (10.4)	
**Country of birth** (M: 26)	**Italy**		5185 (96.0)	1257 (96.7)	3928 (95.8)	0.361
	**Other EU country**		106 (2.0)	21 (1.6)	85 (2.1)	
	**Extra-EU country**		109 (2.0)	22 (1.7)	87 (2.1)	
**Education** (M: 153)	**Junior secondary**		326 (6.2)	37 (2.9)	289 (7.2)	<0.001
	**Secondary**		1512 (28.6)	273 (21.4)	1239 (31.0)	
	**University**		2242 (42.5)	577 (45.2)	1665 (41.6)	
	**Postgraduate**		1199 (22.7)	390 (30.5)	809 (20.2)	
**Ethnicity** (M: 603)	**Caucasian**		4295 (88.9)	1103 (91.9)	3192 (88.0)	<0.001
	**African**		13 (0.3)	4 (0.3)	9 (0.3)	
	**Asian**		9 (0.2)	2 (0.2)	7 (0.2)	
	**Arab**		7 (0.1)	4 (0.3)	3 (0.1)	
	**Latino**		414 (8.6)	75 (6.3)	339 (9.2)	
	**Other**		91 (1.9)	12 (1.0)	79 (2.2)	
**BMI** (M: 231)	**<18**		136 (2.6)	6 (0.5)	130 (3.3)	<0.001
	**18–25**		3391 (65.2)	721 (56.9)	2670 (67.9)	
	**26–30**		1177 (22.6)	419 (33.1)	758 (19.3)	
	**31+**		497 (9.6)	121 (9.4)	376 (9.5)	
**Smoking** (M: 39)	**Never smoker**		3404 (63.1)	765 (58.9)	2639 (64.5)	0.001
	**Smoker**		975 (18.1)	273 (21.0)	702 (17.2)	
	**Ex-smoker**		1014 (18.8)	261 (20.1)	753 (18.4)	
**Years of smoking** **(number)** (M: 37) (restricted to current smokers)	**Mean ± SD**		19.0 ± 11.6	17.3 ± 11.4	19.8 ± 11.6	
	**Median (IQR)**		20 (10; 30)	15 (10; 25)	20 (10; 30)	0.002
	**<6**		135 (14.3)	43 (16.0)	92 (13.7)	0.001
	**6–10**		194 (20.7)	60 (22.3)	134 (19.9)	
	**11–20**		267 (28.3)	93 (34.6)	174 (25.8)	
	**21+**		346 (36.7)	73 (27.1)	273 (40.6)	
**Cigarettes smoked** **(daily number)** (M: 235) (restricted to current smokers)	**Mean ± SD**		8.1 ± 5.9	8.9 ± 7.8	7.8 ± 5.0	
	**Median (IQR)**		7 (4; 10)	8.5 (4; 12)	7 (4; 10)	0.135
	**<5**		196 (26.4)	54 (26.7)	142 (26.3)	0.022
	**5–10**		405 (54.5)	97 (48.0)	308 (56.9)	
	**11+**		142 (19.1)	51 (15.3)	91 (16.8)	
**Years since smoking cessation** **(number)** (M: 72) (restricted to ex-smokers)	**Mean ± SD**		12.7 ± 10.3	11.9 ± 10.7	13.0 ± 10.1	
	**Median (IQR)**		10 (4; 21)	8 (3; 17)	11 (4; 21)	0.062
	**<5**		273 (28.3)	76 (30.8)	197 (27.5)	0.010
	**5–10**		211 (21.9)	68 (27.5)	143 (19.8)	
	**11–20**		237 (24.6)	45 (18.2)	192 (26.9)	
	**21+**		243 (25.2)	58 (23.5)	185 (25.8)	
**Any pre-existing ** **comorbidities**	**No**		3922 (74.2)	982 (76.5)	2940 (73.4)	0.030
	**Yes**		1366 (25.8)	302 (23.5)	1064 (26.6)	
**Immune-depression**	**No**		5309 (97.7)	1282 (97.1)	4027 (97.7)	0.723
	**Yes**		123 (2.3)	28 (2.9)	95 (2.3)	
**Job task** (M:25)	**Medical consultant**		681 (12.6)	302 (23.4)	279 (7.0)	<0.001
	**Medical trainee**		536 (9.9)	194 (15.1)	342 (8.6)	
	**Nurse**		1889 (35.1)	261 (20.3)	1628 (40.8)	
	**Laboratorist**		158 (2.9)	20 (1.6)	138 (3.4)	
	**Nurse aid**		527 (9.8)	74 (5.7)	453 (11.3)	
	**Administrative clerk**		519 (9.8)	127 (9.9)	392 (9.8)	
	**Health technician**		263 (4.9)	73 (5.7)	190 (4.8)	
	**Midwife**		44 (0.8)	0	44 (1.1)	
	**Pharmacist**		34 (0.6)	11 (0.9)	23 (0.6)	
	**Psychologist**		53 (0.9)	5 (0.4)	48 (1.2)	
	**Physio-therapist**		104 (1.9)	24 (1.9)	80 (2.0)	
	**Undergraduate student**		228 (4.2)	78 (6.1)	150 (3.7)	
	**Other**		343 (6.4)	120 (9.3)	223 (5.6)	
**Job seniority ** (years) (M: 613)	**<6**		1525 (28.1)	487 (42.6)	1038 (28.2)	<0.001
	**7–17**		1648 (30.4)	265 (23.2)	783 (21.3)	
	**18–29**		1169 (21.6)	224 (19.6)	945 (25.7)	
	**30+**		1077 (19.9)	167 (14.6)	910 (24.8)	
**Employed in ** **COVID-19 unit now**	**No**		4774 (90.3)	1150 (90.8)	3624 (90.2)	0.564
	**Yes**		510 (9.7)	117 (9.2)	393 (9.8)	
**Actual workplace** (M: 182)	**Infectious diseases**		79 (1.5)	16 (1.3)	63 (1.6)	0.008
	**Pneumology**		63 (1.2)	22 (1.7)	41 (1.0)	
	**ICU**		383 (7.3)	87 (6.9)	296 (7.4)	
	**Internal medicine**		384 (7.2)	76 (6.1)	308 (7.7)	
	**Surgical ward**		592 (11.3)	135 (10.8)	457 (11.4)	
	**Radiology**		147 (2.8)	48 (3.8)	99 (2.5)	
	**Administrative services**		338 (6.4)	96 (7.7)	242 (6.1)	
	**Other**		3264 (62.3)	775 (61.7)	2489 (62.3)	
**Workplace during** **2020–2022** (M: 160)	**Administrative**		679 (12.9)	190 (15.2)	489 (12.2)	<0.001
	**Outpatient**		519 (9.8)	90 (7.2)	429 (10.7)	
	**COVID-19 unit**		46 (0.9)	10 (0.8)	36 (0.9)	
	**Non-COVID-19 unit**		918 (17.4)	168 (13.4)	750 (18.7)	
	**COVID-19 unit (low risk)**		432 (8.2)	87 (6.9)	345 (8.0)	
	**COVID-19 unit (high risk)**		425 (8.1)	97 (7.7)	328 (8.2)	
	**Operating theatre**		474 (9.0)	185 (14.8)	289 (7.2)	
	**Other**		1779 (33.8)	426 (34.0)	1353 (33.7)	

**Table 2:** COVID-19 infections (1st, 2nd, and 3rd events) in healthcare workers by explanatory factors. Number (N); column percentage (%); mean ± standard deviation (SD); and median with interquartile rage (IQR). M = missing.

Terms	Strata		COVID-19 Infections		
			Only One (N = 2163)	Only Two (N = 230)	Three (N = 8)
**Center**	**Verona**		1063 (49.1)	122 (53.0)	3 (37.5)
	**Padua**		594 (27.5)	45 (19.6)	2 (25.0)
	**Trieste**		381 (17.6)	50 (21.7)	1 (12.5)
	**Modena-Reggio Emilia**		125 (5.8)	13 (5.7)	2 (25.0)
**Doses of ** **COVID-19 vaccine before infection**	**0**		246 (11.4)	52 (22.6)	1 (12.5)
	**1**		88 (4.1)	17 (7.4)	0
	**2**		205 (9.5)	43 (18.7)	4 (50.0)
	**3**		1623 (75.0)	118 (51.3)	3 (37.5)
	**4**		1 (0.1)	0	0
**COVID-19** **wave**	**1 March 2020–31 October 2020**		153 (7.2)	0	0
	**1 November 2020–31 May 2021**		291 (13.6)	14 (6.0)	0
	**1 June 2021–30 November 2021**		69 (3.1)	6 (2.6)	0
	**1 December 2021–25 August 2022**		1622 (76.0)	215 (91.9)	8 (14.3)
**Time between last dose of COVID-19 vaccine and SARS-CoV-2 infection ** (days)	**Any dose**	**M ± SD**	136.3 ± 85.6	152.6 ± 111.6	143.86 ± 87.0
		**M (IQR)**	122 (74; 178)	127 (75; 205)	121 (65; 227)
	**1st dose**	**M ± SD**	344.2 ± 130.3	351.9 ± 125.1	NA
		**M (IQR)**	381 (245; 451)	366 (276; 451)	NA
	**2nd dose**	**M ± SD**	244.5 ± 119.9	167 ± 122.9	172 ± 71.7
		**M (IQR)**	254 (150; 344)	139.5 (62.5; 244.5)	170.5 (106; 239.5)
	**3rd dose**	**M ± SD**	121.1 ± 61.3	117.7 ± 60.5	106.3 ± 106.2
		**M (IQR)**	118 (70; 167)	106 (73; 153)	65 (27; 227)
	**4th dose**	**M ± SD**	NA	NA	NA
		**M (IQR)**	NA	NA	NA
**Viral shedding ** **time ** (days)	**Mean ± SD**		13.5 ± 8.0	10.0 ± 4.5	9.3 ± 5.7
	**Median (IQR)**		11 (9; 15)	8.5 (7; 12)	7 (7; 10.5)
	**0–7**		431 (21.2)	91 (42.05)	5 (83.3)
	**8–10**		560 (27.5)	64 (29.9)	0
	**11–14**		402 (19.8)	23 (10.8)	0
	**15+**		642 (31.6)	36 (16.8)	1 (16.7)
**Hospitalization**	**No**		2146 (99.2)	229 (99.6)	8 (100)
	**Yes**		17 (0.8)	1 (0.4)	0
**Length of stay ** (days)	**Mean ± SD**		6.9 ± 5.1	1	NA
	**Median (IQR)**		6.5 (2; 10)	1	NA
	**<6**		7 (43.8)	1	NA
	**6–10**		6 (37.5)	0	NA
	**11–15**		3 (18.8)	0	NA
	**16+**		0	0	NA

**Table 3:** Distribution of COVID-19 hospitalizations by pandemic wave. Number and column percentage.

Term	Strata	Wuhan	Alpha	Delta	Omicron
**Admission after ** **1st COVID-19 infection**	**No**	207 (95.4)	387 (99.5)	79 (98.5)	1638 (99.6)
	**Yes**	10 (4.6)	2 (0.5)	2 (2.5)	6 (0.4)

**Table 4:** Counts of the individual, combined, and total symptoms following a first COVID-19 event. Number and percentage (%).

Post-COVID-19 Symptoms		Number	(%)
**Single** **symptoms**	Asthenia	126	14.2
	Cough	79	8.9
	Myalgia	27	3.0
	Dyspnea	18	2.0
	Anosmia	16	1.8
	Concentration deficit	13	1.5
	Headache	12	1.4
	Dysgeusia	9	1.0
**2 symptoms** **combined**	Asthenia, myalgia	53	6.0
	Asthenia, cough	42	4.7
	Asthenia, dyspnea	34	3.8
	Anosmia, ageusia	26	2.9
	Asthenia, headache	23	2.6
	Asthenia, concentration deficit	21	2.4
**3 symptoms** **combined**	Asthenia, myalgia, dyspnea	14	1.6
	Asthenia, myalgia, cough	11	1.2
	Asthenia, anosmia, dysgeusia	10	1.1
	Asthenia, myalgia, concentration deficit	10	1.1
	Asthenia, myalgia, headache	10	1.1
**Total** **symptom** **count**	Asthenia	596	30.3
	Myalgia	270	13.7
	Cough	244	12.4
	Dyspnea	200	10.2
	Concentration deficit	160	8.1
	Headache	143	7.3
	Anosmia	128	6.5
	Dysgeusia	113	5.7
	Insomnia	102	5.2
	Anxiety	84	4.3

**Table 5:** Spearman correlation and *p*-value * ** between the most frequent symptoms following a first negative swab after a 1st or 2nd COVID-19 episode. Heat-map: light orange highlights mark significant correlations (*p* < 0.01); darker orange highlights mark highly significant correlations (*p* < 0.001).

	Symptoms	Asthenia	Myalgia	Cough	Dyspnea	Concentration Deficit	Headache	Anosmia	Dysgeusia	Insomnia
**1st COVID-19 infection**	**Asthenia**	1	0.412 **	0.182 **	0.328 **	0.321 **	0.238 **	0.098 **	0.121 **	0.241 **
	**Myalgia**		1	0.073 **	0.247 **	0.215 **	0.225 **	0.060 *	0.091 **	0.190 **
	**Cough**			1	0.123 **	0.060 *	0.093 **	0.069 *	0.022	0.059 *
	**Dyspnea**				1	0.162 **	0.165 **	0.065 *	0.149 **	0.147 **
	**Concentration deficit**					1	0.185 **	0.060 *	0.035	0.276 **
	**Headache**						1	0.058 *	0.084 **	0.225 **
	**Anosmia**							1		0.045 *
	**Dysgeusia**								1	0.072 **
	**Insomnia**									1
**2nd COVID-19 infection**	**Asthenia**	1	-	0.247 **	0.034	0.107	0.187 *	0.003	0.268 **	0.125
	**Myalgia**		1	-	-	-	-	-	-	-
	**Cough**			1	−0.027	−0.008	−0.015	−0.017	−0.015	−0.013
	**Dyspnea**				1	0.143	0.151	0.204 *	−0.047	−0.043
	**Concentration deficit**					1	−0.015	0.238 **	−0.015	−0.013
	**Headache**						1	0.119	−0.026	0.164 *
	**Anosmia**							1	−0.030	0.164 *
	**Dysgeusia**								1	0.027
	**Insomnia**									1

* Spearman correlation *p* < 0.01 ** Spearman correlation *p* < 0.001.

**Table 6:** Symptoms developed during acute disease (1st COVID-19 infection) and/or present afterward (persisting since acute COVID-19 or newly developed after a first negative swab). Number (N); percentage (%).

Strata	Symptoms during Acute COVID-19		Overall Post-COVID-19 Symptoms						Persisting Symptoms *		Novel Symptoms **	
			All		At 30–60 days		At 61+ days					
	N	%	N	%	N	%	N	%	N	%	N	%
**General**	1526/1969	77.5	692/1969	35.1	344/692	49.7	226/691	32.7	639/1526	41.9	79/443	17.8
**ENT**	1393/1969	70.8	319/1969	16.2	148/319	46.4	98/317	30.9	282/1393	20.2	51/576	8.9
**Pulmonary**	1067/1969	54.2	413/1969	21.0	187/413	45.3	109/413	26.4	377/1067	35.3	44/902	4.9
**Gastro-enteric**	486/1969	24.7	58/1969	2.4	28/58	48.3	17/58	29.3	51/486	10.5	12/1483	0.8
**Psychological**	325/1969	16.5	167/1969	8.4	115/167	68.9	76/167	45.5	127/325	39.1	47/1644	2.9
**Neurological**	326/1969	16.6	210/1969	10.7	142/210	67.6	99/209	47.4	150/326	46.0	80/1643	4.9

* persisting since acute COVID-19, after first negative swab test; ** newly developed after first negative swab test, but not present during acute COVID-19.

**Table 7:** Presence of any symptoms at 30–60 days or 61+ days since a first negative swab test after a primary COVID-19 infection, by explanatory variable. Univariable logistic regression analysis. Number (N), row percentage (%), and odds ratio (OR) with a 95% confidence interval (95%CI).

Term	Strata	1st COVID-19 Infection (N = 2401) N (%)	Long COVID-19 after Primary Infection					
			30–60 Days Since First Negative Swab			61+ Days Since First Negative Swab		
			No (N = 1825) N (%)	Yes (N = 576) N (%)	OR (95%CI)	No (N = 2005) N (%)	Yes (N = 389) N (%)	OR (95%CI)
**Center**	**Verona**	1188 (49.5)	904 (76.1)	284 (23.9)	*reference*	993 (83.8)	192 (16.2)	*reference*
	**Padua**	641 (26.7)	483 (75.4)	158 (24.6)	1.04 (0.83; 1.30)	542 (84.6)	99 (15.4)	0.94 (0.73; 1.23)
	**Trieste**	431 (18.0)	311 (72.0)	121 (28.0)	1.24 (0.97; 1.59)	337 (78.7)	91 (21.3)	1.39 (1.06; 1.84)
	**Modena-Reggio Emilia**	140 (5.8)	127 (90.7)	13 (9.3)	0.33 (0.18: 0.59)	133 (95.0)	7 (5.0)	0.27 (0.13; 0.59)
**COVID-19** **wave**	**1 March 2020–31 October 2020**	291 (9.3)	115 (52.5)	104 (47.5)	4.77 (3.00; 7.71)	94 (63.1)	55 (36.9)	6.31 (4.09; 9.75)
	**1 November 2020–31 May 2021**	393 (16.7)	229 (58.3)	164 (41.7)	3.78 (2.88; 5.00)	266 (64.9)	144 (35.1)	4.43 (3.30; 5.94)
	**1 June 2021–30 November 2021**	82 (3.5)	59 (72.0)	23 (15.9)	2.06 (1.05; 4.04)	110 (81.6)	25 (18.5)	2.60 (1.17; 5.80)
	**1 December 2021–25 August 2022**	1665 (70.6)	1400 (84.1)	265 (16.0)	*reference*	1513 (90.9)	152 (9.2)	*reference*
**SARS-CoV-2 p4ositivity** (days)	**0–7**	444 (19.6)	397 (89.4)	47 (10.6)	*reference*	419 (94.4)	25 (5.6)	*reference*
	**8–10**	604 (26.7)	502 (83.1)	102 (16.9)	1.72 (1.17; 2.52)	543 (90.4)	58 (9.7)	1.79 (0.91; 3.54)
	**11–14**	431 (19.1)	343 (79.6)	88 (20.4)	2.17 (1.49; 3.16)	381 (88.6)	49 (11.4)	2.16 (0.99; 4.68)
	**15+**	783 (35.6)	469 (59.9)	314 (40.1)	5.66 (5.21; 6.14)	542 (69.5)	238 (30.5)	7.36 (5.36; 10.12)
**Hospitalization**	**No**	2380 (99.1)	1816 (76.3)	564 (23.7)	*reference*	1995 (84.0)	379 (16.1)	*reference*
	**Yes**	21 (0.9)	9 (42.9)	12 (57.1)	4.28 (1.48; 12.39)	10 (50.0)	10 (50.0)	5.25 (2.50; 11.00)
**Vaccination status** (number of doses before 1st infection)	**0**	827 (34.7)	504 (60.9)	323 (39.1)	*reference*	583 (70.5)	244 (29.5)	*reference*
	**1**	90 (3.8)	76 (84.4)	14 (15.6)	0.29 (0.17; 0.50)	81 (90.0)	9 (10.0)	0.27 (0.15; 0.48)
	**2**	206 (8.6)	166 (80.6)	40 (19.4)	0.38 (0.29; 0.49)	179 (86.9)	27 (13.1)	0.36 (0.25; 0.53)
	**3**	1263 (52.9)	1069 (84.6)	194 (15.4)	0.28 (0.20; 0.40)	1156 (91.5)	107 (8.5)	0.22 (0.15; 0.33)
	**4**	0	NA	NA	NA	0	1	NA
**Sex**	**Male**	535 (22.1)	453 (84.7)	82 (15.3)	*reference*	488 (91.2)	47 (8.8)	*reference*
	**Female**	1866 (77.7)	1372 (73.5)	484 (26.5)	1.99 (1.67; 2.36)	1517 (81.6)	342 (18.4)	2.34 (1.75; 3.14)
**Age** (years)	**Mean ± SD**	44.7 ± 12.0	42.6 ± 11.7	46.7 ± 10.4	NA	42.6 ± 11.7	46.7 ± 10.4	NA
	**Median (IQR)**	47 (33; 55)	43 (31; 53)	49 (39; 55)	*p* < 0.001	43 (31; 53)	49 (39; 55)	*p* < 0.001
	**<40**	860 (41.0)	728 (84.7)	132 (15.4)	*reference*	771 (89.8)	88 (10.2)	*reference*
	**40–54**	798 (38.0)	562 (70.4)	236 (29.6)	2.32 (1.72; 3.12)	634 (79.5)	164 (20.6)	2.27 (1.74; 2.96)
	**55+**	441 (21.0)	311 (70.5)	130 (29.5)	2.30 (1.95; 2.73)	352 (80.2)	87 (19.8)	2.17 (1.74; 2.69)
**Country ** **of birth**	**Italy**	2300 (96.2)	1756 (76.4)	544 (23.7)	*reference*	1926 (84.0)	367 (16.0)	*reference*
	**EU**	45 (1.9)	34 (75.6)	11 (24.4)	1.04 (0.31; 3.47)	35 (77.8)	10 (22.2)	1.50 (0.49; 4.57)
	**Extra-EU**	46 (1.9)	28 (60.9)	18 (39.1)	2.08 (1.13; 3.81)	36 (78.3)	10 (21.7)	1.46 (1.16; 1.84)
**Ethnicity**	**Caucasian**	1912 (89.1)	1492 (78.0)	420 (22.0)	*reference*	1631 (85.3)	276 (14.5)	*reference*
	**Other**	235 (11.0)	166 (70.6)	69 (29.4)	1.43 (1.24; 1.65)	179 (76.5)	55 (23.5)	1.82 (1.60; 2.06)
**Educational** **level**	**Junior secondary**	143 (6.1)	88 (61.5)	55 (38.5)	*reference*	105 (73.9)	37 (25.5)	*reference*
	**Secondary**	612 (26.1)	441 (72.1)	171 (27.9)	0.63 (0.46; 0.87)	485 (79.5)	125 (20.5)	0.73 (0.66; 0.81)
	**University**	1083 (46.2)	860 (79.4)	223 (20.6)	0.41 (0.32 0.59)	935 (86.6)	145 (13.7)	0.44 (0.33; 0.59)
	**Postgraduate**	505 (21.6)	397 (78.6)	108 (21.4)	0.48 (0.42; 0.52)	438 (86.9)	66 (13.4)	0.43 (0.40; 0.46)
**Marital** **Status**	**Single**	689 (29.1)	557 (80.8)	132 (19.2)	*reference*	605 (87.7)	86 (12.1)	*reference*
	**Married**	1096 (46.2)	805 (73.5)	291 26.6)	1.54 (1.12; 2.12)	898 (81.6)	202 (18.4)	1.60 (0.91; 2.80)
	**Cohabitant**	363 (15.3)	299 (82.4)	64 (17.6)	0.89 (0.69; 1.13)	318 (87.6)	45 (12.4)	1.01 (0.77; 1.32)
	**Divorced/separated/widow**	191 (8.1)	120 (62.8)	71 (37.2)	2.49 (2.09; 2.97)	147 (76.2)	46 (23.8)	2.23 (1.74; 2.85)
**BMI** (kg/m^2^)	**<18**	53 (2.3)	38 (67.9)	18 (32.1)	1.72 (0.80; 3.67)	47 (83.9)	9 (17.0)	1.18 (0.45; 3.12)
	**18–25**	1523 (65.8)	1251 (78.4)	345 (21.6)	*reference*	1370 (86.1)	222 (13.9)	*reference*
	**26–30**	521 (22.5)	393 (71.7)	155 (28.3)	1.43 (1.10; 1.90)	435 (79.7)	111 (20.3)	1.57 (1.27; 1.96)
	**31+**	218 (9.4)	142 (63.4)	82 (36.6)	2.09 (1.24; 3.52)	167 (74.6)	57 (25.5)	2.11 (1.22; 3.64)
**Any pre-existing** **conditions**	**No**	2034 (38.4)	767 (73.5)	276 (26.5)	*reference*	857 (82.5)	182 817.5)	*reference*
	**Yes**	3263 (61.6)	1075 (76.3)	334 (23.7)	0.86 (0.65; 1.15)	1182 (84.1)	224 (15.9)	0.89 (0.64; 1.25)
**Under psychological** **therapy**	**No**	2313 (97.8)	1761 (76.1)	552 (23.9)	*reference*	1934 (83.9)	372 (16.1)	*reference*
	**Yes**	51 (2.2)	35 (68.6)	16 (31.4)	1.46 (0.94; 2.27)	38 (74.5)	13 (25.5)	1.78 (0.89; 3.57)
**Smoking** **status**	**Never smoked**	1509 (63.3)	1170 (77.5)	339 (22.5)	*reference*	1278 (84.9)	227 (15.1)	*reference*
	**Smoker**	402 (16.9)	301 (74.9)	101 (25.1)	1.16 (0.85; 1.58)	337 (84.0)	64 (16.0)	1.07 (0.81; 1.41)
	**Ex- smoker**	473 (19.8)	342 (72.3)	131 (27.7)	1.32 (1.16; 1.50)	378 (80.2)	93 (19.8)	1.39 (1.17; 1.64)
**Years of** **smoking** (Number)	**Mean ± SD**	17.1 ± 11.0	16.2 ± 11.2	19.3 ± 10.3	NA	16.5 ± 11.0	20.0 ± 10.5	NA
	**Median (IQR)**	15 (8; 25)	15 (8; 25)	20 (10; 28)	0.008	15 (8; 25)	20 (10; 30)	0.016
	**<6**	70 (18.0)	60 (85.7)	10 (14.3)	*reference*	64 (92.8)	5 (7.2)	*reference*
	**6–10**	88 (22.7)	69 (78.4)	19 (21.6)	1.65 (1.10; 2.48)	75 (85.2)	13 (14.8)	2.22 (1.08; 4.54)
	**11–20**	114 (29.4)	83 (72.8)	31 (27.2)	2.24 (1.51; 3.34)	93 (81.6)	21 (18.4)	2.89 (1.55; 5.40)
	**21+**	116 (29.9)	81 (69.8)	35 (30.2)	2.59 (1.94; 3.46)	94 (81.0)	22 (19.0)	3.00 (1.46; 6.14)
**Cigarettes** **smoked** (daily number)	**Mean ± SD**	7.2 ± 4.8	7.1 ± 4.9	7.4 ± 4.6	NA	7.2 ± 4.8	7.2 ± 4.6	NA
	**Median (IQR)**	6 (3; 10)	6 (3; 10)	6 (3; 10)	0.914	6 (3; 10)	6 (3; 10)	0.480
	**<5**	97 (32.1)	79 (81.4)	18 (18.6)	*reference*	85 (87.6)	12 (12.5)	*reference*
	**5–10**	161 (53.3)	121 (75.2)	40 (24.8)	1.45 (1.02; 2.06)	137 (85.1)	24 (15.0)	1.24 (0.81; 1.91)
	**11+**	44 (14.6)	35 (79.6)	9 (20.5)	1.13 (0.54; 2.36)	39 (88.6)	5 (11.6)	0.91 (0.19; 4.41)
**Years since** **smoking cessation** (Number)	**Mean ± SD**	11.5 ± 9.4	11.3 ± 9.3	13.3 ± 9.6	NA	11.0 ± 9.4	13.3 ± 9.4	NA
	**Median (IQR)**	9.5 (3; 19)	9 (3; 18)	12 (4.5; 22)	0.031	8 (3; 18)	11.5 (5.5; 21.5)	0.026
	**<5**	145 (31.5)	112 (77.2)	33 (22.8)	*reference*	125 (86.2)	20 (13.8)	*reference*
	**5–10**	103 (22.4)	80 (77.7)	23 (22.3)	0.98 (0.54; 1.75)	86 (84.3)	16 (15.7)	1.16 (0.49; 2.77)
	**11–20**	120 (26.1)	85 (70.8)	35 (29.2)	1.40 (1.04;1.87)	94 (78.3)	26 (21.7)	1.73 (0.90; 3.31)
	**21+**	92 (20.0)	60 (65.2)	32 (34.8)	1.81 (1.27; 2.58)	69 (75.8)	22 (24.2)	1.99 (0.84; 4.73)
**Current workplace**	**Infectious diseases**	45 (1.9)	35 (77.8)	10 (22.2)	0.55 (0.40; 0.75)	37 (84.1)	7 /15.9)	0.66 (0.51; 0.86)
	**Pneumology**	36 (1.5)	32 (88.9)	4 (11.1)	0.24 (0.06; 0.95)	32 (91.4)	3 (8.6)	0.33 (0.06; 1.70)
	**ICU**	183 (7.8)	139 (76.0)	44 (24.0)	0.61 (0.41; 0.91)	147 (80.3)	36 (19.7)	0.86 (0.48; 1.52)
	**Internal medicine**	199 (8.5)	153 (76.9)	46 (23.1)	0.58 (0.33; 1.03)	167 (83.9)	32 (16.1)	0.67 (0.48; 0.93)
	**Surgical ward**	293 (12.5)	218 (74.4)	75 (25.6)	0.66 (0.49; 0.89)	250 (85.3)	43 (14.7)	0.60 (0.37; 0.97)
	**Radiology**	54 (2.3)	40 (74.1)	14 (25.9)	0.67 (0.36; 1.27)	44 (81.5)	10 (18.5)	0.80 (0.47; 1.33)
	**Administrative services**	117 (5.0)	77 (65.8)	40 (34.2)	*Reference*	91 (77.8)	26 (22.2)	*reference*
	**Other**	1415 (60.4)	1080 (76.3)	335 (23.7)	0.60 (0.49; 0.72)	1185 (84.0)	225 (16.0)	0.66 (0.50; 0.88)
**Employed in ** **COVID-19 unit now**	**No**	2056 (88.4)	1575 (76.6)	481 (23.4)	*reference*	1731 (84.2)	325 (15.8)	*reference*
	**Yes**	269 (11.6)	191 (71.0)	78 (29.0)	1.34 (1.00; 1.79)	216 (80.3)	53 (19.7)	1.31 (1.17; 1.46)
**Job task**	**Medical consultant**	282 (11.8)	224 (79.4)	58 (20.6)	0.57 (0.30; 1.08)	249 (88.3)	33 (11.7)	0.44 (0.31; 0.61)
	**Medical trainee**	276 (11.6)	253 (91.7)	23 (8.3)	0.20 (0.15; 0.26)	261 (94.9)	14 (5.1)	0.18 (0.13; 0.24)
	**Nurse**	923 (38.8)	655 (71.0)	268 (29.0)	0.90 (0.67; 1.21)	735 (79.8)	186 (20.2)	0.83 (0.63; 1.10)
	**Laboratorist**	71 (3.0)	60 (84.5)	11 (15.5)	0.40 (0.19; 0.87)	65 (91.6)	6 (8.5)	0.30 (0.12; 0.80)
	**Nurse aid**	245 (10.3)	160 (65.3)	85 (34.7)	1.17 (0.73; 1.86)	187 (76.3)	58 (23.7)	1.02 (0.56; 1.87)
	**Administrative clerk**	182 (7.6)	125 (68.7)	57 (31.3)	*reference*	138 (76.7)	42 (23.3)	*reference*
	**Health technician**	107 (4.5)	88 (82.2)	19 (17.8)	0.47 (0.32; 0.69)	94 (87.9)	13 (12.2)	0.45 (0.23; 0.88)
	**Midwife**	24 (1.0)	20 (83.3)	4 (16.7)	0.44 (0.18; 1.06)	21 (87.5)	3 (12.5)	0.47 (0.21; 1.05)
	**Pharmacist**	15 (0.6)	13 (86.7)	2 (13.3)	0.34 (0.07; 1.73)	14 (93.3)	1 (6.7)	0.23 (0.05; 1.19)
	**Psychologist**	28 (1.2)	23 (82.1)	5 (17.9)	0.48 (0.33; 0.69)	24 (85.7)	4 (14.3)	0.55 (0.20; 1.47)
	**Physio-therapist**	47 (2.0)	39 (83.0)	8 (17.0)	0.45 (0.24; 0.85)	41 (91.1)	4 (8.9)	0.32 (0.13; 0.79)
	**Undergraduate student**	62 (2.6)	58 (93.6)	4 (6.5)	0.15 (0.12; 0.18)	58 (93.6)	4 (6.5)	0.23 (0.19; 0.26)
	**Other**	120 (5.0)	94 (78.3)	25 (21.7)	0.61 (0.44; 0.83)	100 (83.3)	20 (16.5)	0.66 (0.42; 1.02)
**Job seniority** (years)	**<6**	749 (34.6)	631 (84.3)	118 (15.8)	*reference*	674 (90.1)	74 (9.9)	*reference*
	**7–17**	484 (22.4)	354 (73.1)	130 (26.9)	1.96 (1.48; 2.61)	399 (82.4)	85 (17.6)	1.94 (1.41; 2.68)
	**18–29**	530 (24.5)	365 (68.9)	165 (31.1)	2.42 (1.95; 2.99)	415 (78.6)	113 (21.4)	2.48 (1.68; 3.67)
	**30+**	401 (18.5)	287 (71.6)	114 (28.4)	2.12 (1.66; 2.72)	313 (78.8)	84 (21.2)	2.44 (1.59; 3.76)
**Workplace during** **2020–2022**	**Administrative**	236 (10.1)	168 (71.2)	68 (28.8)	*reference*	193 (81.8)	43 (18.2)	*reference*
	**Outpatient**	226 (9.6)	173 (76.6)	53 (23.5)	0.76 (0.52; 1.10)	187 (83.5)	37 (16.5)	0.89 (0.62; 1.26)
	**COVID-19 unit**	19 (0.8)	13 (68.4)	6 (31.6)	1.14 (0.48; 2.73)	14 (73.7)	5 (26.3)	1.60 (0.54; 4.76)
	**Non-COVID-19 unit**	462 (19.7)	354 (76.6)	108 (23.4)	0.75 (0.51; 1.12)	391 (84.6)	71 (15.4)	0.82 (0.55; 1.20)
	**COVID-19 unit (low risk)**	231 (9.8)	168 (72.7)	63 (27.3)	0.93 (0.58; 1.48)	192 (83.5)	38 (16.5)	0.89 (0.72; 1.09)
	**COVID-19 unit (high risk)**	206 (8.8)	160 (77.7)	46 (22.3)	0.71 (0.36; 1.41)	166 (80.6)	40 (19.4)	1.08 (0.59; 1.97)
	**Operating theatre**	209 (8.9)	173 (82.8)	36 (17.2)	0.51 (0.35; 0.76)	191 (91.8)	17 (8.2)	0.40 (0.24; 0.65)
	**Other**	758 (32.3)	572 (75.5)	183 (24.5)	0.80 (0.59; 1.09)	625 (82.8)	130 (17.0)	0.93 (0.68; 1.27)

**Table 8:** Multiple logistic regression analysis for the risk of long COVID-19 at 30–60 days or 61+ days since first a negative swab after a **primary COVID-19 event**. Adjusted odds ratio (aOR) with 95% confidence interval (95%CI). Heat-map: orange color highlights increased risk (OR > 1); green color highlights reduced risk (OR < 1).

Term	Strata	Long COVID-19 after Primary Infecion (aOR; 95%CI)	
		30–60 Days Since 1st Negative Swab (1602 Obs.)	61+ Days Since 1st Negative Swab (1611 Obs.)
**Sex**	**Male**	*reference*	*reference*
	**Female**	1.91 (1.30; 2.80)	2.14 (1.69; 2.71)
**Age ** (years)	**<40**	*reference*	*Reference*
	**40–54**	1.73 (1.25; 2.39)	1.69 (1.47; 1.93)
	**55+**	1.60 (1.45; 1.76)	1.60 (1.24; 2.06)
**COVID-19** **wave**	**1 March 2020–31 October 2020**	2.16 (1.14; 4.09)	2.25 (1.00; 5.09)
	**1 November 2020–31 May 2021**	2.05 (1.25; 3.38)	1.91 (0.86; 4.21)
	**1 June 2021–30 November 2021**	1.68 (0.72; 3.93)	2.18 (0.71; 6.68)
	**1 December 2021–25 August 2022**	*reference*	*reference*
**Viral shedding time** (days)	**0–7**	*reference*	*reference*
	**8–10**	1.68 (1.11; 2.53)	2.03 (1.02; 4.04)
	**11–14**	1.92 (1.49; 2.48)	1.97 (1.46; 2.67)
	**15+**	3.20 (2.07; 4.94)	5.00 (2.84; 8.81)
**COVID-19 hospitalization**	**No**	*reference*	*reference*
	**Yes**	3.34 (1.62; 6.89)	4.09 (2.25; 7.44)
**COVID-19 vaccination status before infection** (number of doses)	**0**	*reference*	*reference*
	**1**	0.83 (0.54; 1.27)	0.60 (0.36; 1.36)
	**2**	0.57 (0.34; 0.94)	0.60 (0.36; 0.99)
	**3**	0.77 (0.51; 1.17)	0.71 (0.44; 1.14)
**Ethnicity**	**Caucasian**	*reference*	*reference*
	**Other**	1.21 (0.97; 1.51)	1.83 (1.41; 2.36)
**Educational** **level**	**Junior secondary**		*reference*
	**Secondary**		0.73 (0.23; 2.24)
	**University**		0.67 (0.24; 1.89)
	**Postgraduate**		0.56 (0.19; 1.71)
**Marital** **status**	**Single**	*reference*	
	**Married**	0.91 (0.67; 1.22)	
	**Cohabitant**	0.70 (0.47; 1.05)	
	**Divorced/separated/widow**	1.22 (1.15; 1.30)	
**BMI** **(Kg/m^2^)**	**<18**	2.52 (1.29; 4.92)	1.40 (0.33; 5.93)
	**18–25**	*reference*	*reference*
	**26–30**	1.43 (1.18; 1.73)	1.72 (1.29; 2.29)
	**31+**	1.68 (1.48; 1.91)	1.56 (1.26; 1.92)
**Job task** (M: 41)	**Medical consultant**	1.23 (0.76; 2.01)	0.81 (0.55; 1.19)
	**Medical trainee**	0.74 (0.59; 0.93)	0.47 (0.29; 0.77)
	**Nurse**	1.31 (0.95; 1.79)	0.86 (0.48; 1.55)
	**Laboratorist**	0.67 (0.32; 1.43)	0.37 (0.21; 0.67)
	**Nurse aid**	1.10 (0.79; 1.54)	0.53 (0.38; 0.72)
	**Administrative clerk**	*reference*	*reference*
	**Health technician**	0.86 (0.68; 1.09)	0.73 (0.59; 0.91)
	**Midwife**	0.62 (0.18; 2.05)	0.37 (0.21; 0.64)
	**Pharmacist**	1.07 (0.14; 8.24)	0.71 (0.17; 2.93)
	**Psychologist**	1.09 (0.93; 1.27)	1.40 (0.78; 2.51)
	**Physio-therapist**	1.25 (0.81; 1.93)	0.40 (0.13; 1.26)
	**Undergraduate student**	0.27 (0.19; 0.40)	0.72 (0.41; 1.25)
	**Other**	1.15 (0.84; 1.57)	1.31 (0.58; 2.95)
**Current workplace** (M: 59)	**Infectious diseases**	0.46 (0.12; 1.76)	1.74 (0.21; 14.54)
	**Pneumology**	0.38 (0.08; 1.87)	0.90 (0.10; 8.31)
	**ICU**	0.71 (0.39; 1.31)	1.75 (0.51; 6.01)
	**Internal medicine**	0.39 (0.24; 0.62)	0.78 (0.44; 1.39)
	**Surgical ward**	0.52 (0.36; 0.77)	0.69 (0.15; 3.30)
	**Radiology**	1.05 (0.51; 2.19)	2.25 (0.75; 6.78)
	**Administrative services**	*reference*	*reference*
	**Other**	0.64 (0.52; 0.78)	1.20 (0.47; 3.06)

**Table 9:** Univariable and multivariable logistic regression analysis for the risk of long COVID-19 30–60 days or 61+ days since a first negative swab following a **second** COVID-19 event. Odds ratio unadjusted (OR) and adjusted (aOR), with a 95% confidence interval (95%CI).

Term	Strata	2nd COVID-19 Infection (N = 238) N (%)	Long COVID-19 at 30–60 Days Since 1st Negative Swab				Long COVID-19 at 61+ Days Since 1st Negative Swab			
			No (N = 213) N (%)	Yes (N = 25) N (%)	Logistic regression		No (N = 225) N (%)	Yes (N = 13) N (%)	Logistic regression	
					Univariable (OR; 95%CI)	Multivariable (aOR; 95%CI) (214 obs.)			Univariable (OR; 95%CI)	Multivariable (aOR; 95%CI) (141 obs.)
**COVID-19 vaccination status before infection**	**0**	44 (18.7)	36 (81.8)	8 (17.4)	*reference*	*reference*	38 (86.4)	6 (13.0)	*reference*	*reference*
	**1**	25 (10.6)	23 (92.0)	2 (8.0)	0.39 (0.10; 1.47)	0.71 (0.16; 3.12)	24 (96.0)	1 (4.0)	0.26 (0.05; 1.49)	0.44 (0.17; 1.11)
	**2**	56 (23.8)	53 (94.6)	3 (5.5)	0.25 (0.06; 1.02)	0.47 (0.08; 2.76)	56 (100)	0	*omitted*	*omitted*
	**3**	110 (46.8)	99 (90.0)	11 (10.0)	0.50 (0.17; 1.50)	0.93 (0.18; 4.90)	105 (95.5)	5 (4.8)	0.30 (0.07; 1.23)	0.40 (0.10; 1.67)
	**4**	0	NA	NA	NA	NA	NA	NA	NA	NA
**Sex**	**Male**	48 (20.2)	42 (87.5)	6 (12.5)	*reference*	*reference*	45 (93.8)	3 (6.3)	*reference*	*reference*
	**Female**	190 (79.8)	171 (90.0)	19 (10.0)	0.78 (0.49; 1.24)	0.77 (0.40; 1.46)	180 (94.7)	10 (5.3)	0.83 (0.44; 1.58)	0.74 (0.31; 1.76)
**Age ** (years)	**<40**	88 (43.1)	83 (94.3)	5 (5.7)	*reference*		86 (97.7)	2 (2.3)	*reference*	
	**40–54**	83 (40.7)	73 (88.0)	10 (12.1)	2.27 (0.80; 6.50)		79 (95.2)	4 (4.8)	2.18 (0.31; 15.16)	
	**55+**	33 (16.2)	29 (87.9)	4 (12.1)	2.29 (0.63; 6.27)		30 (90.9)	3 (9.1)	4.30 (0.66; 27.92)	
**COVID-19** **wave**	**1 March 2020–31 Oct 2020**	0	0	0	*omitted*	*omitted*	0	0	Omitted	Omitted
	**1 Nov 2020–31 May 2021**	14 (6.0)	11 (78.6)	3 (21.4)	2.52 (1.64; 3.86)	1.03 (0.30; 3.57)	12 (85.7)	2 (14.3)	3.42 (0.67; 17.36)	0.54 (0.03; 9.87)
	**1 June 2021–30 Nov 2021**	6 (2.6)	6 (100)	0	1	Omitted	6 (100)	0	Omitted	*Omitted*
	**1 Dec 2021–25 Aug 2022**	215 (91.5)	194 (90.2)	21 (9.8)	*reference*	*reference*	205 (95.4)	10 (4.7)	*reference*	*reference*
**Viral shedding time** (days)	**0–7**	95 (43.0)	90 (94.7)	5 (5.3)	*reference*	*reference*	92 (96.8)	3 (3.2)	*reference*	*reference*
	**8–10**	65 (29.4)	59 (90.8)	6 (9.2)	1.83 (1.25; 2.69)	1.71 (1.51; 1.95)	62 (95.4)	3 (4.6)	1.48 (0.44; 4.97)	1.13 (0.38; 3.30)
	**11–14**	23 (10.4)	22 (95.7)	1 (4.4)	0.82 (0.10; 6.70)	0.78 (0.08; 7.42)	23 (100)	0	*omitted*	*omitted*
	**15+**	38 (17.2)	30 (79.0)	8 (21.1)	4.80 (2.87; 8.02)	4.76 (2.81; 8.07)	35 (92.1)	3 (7.9)	2.63 (1.49; 4.63)	2.30 (0.98; 5.41)
**BMI** (Kg/m^2^)	**<18**	2 (0.9)	2 (100)	0	Omitted		2 (100)	0	Omitted	Omitted
	**18–25**	157 (68.6)	140 (82.1)	12 (7.9)	*reference*		152 (96.8)	5 (3.2)	*reference*	*reference*
	**26–30**	46 (20.1)	37 (80.4)	9 (19.6)	2.94 (0.82; 10.59)		41 (89.1)	5 (10.9)	3.71 (2.81; 4.90)	4.66 (3.54; 6.14)
	**31+**	24 (10.5)	20 (83.3)	4 (16.7)	2.42 (0.28; 21.43)		21 (87.5)	3 (12.5)	4.34 (0.57; 33.01)	1.73 (0.33; 8.97)
**Ethnicity**	**Caucasian**	182 (87.5)	162 (89.0)	20 (11.0)	*reference*	*reference*	45 (93.8)	3 (6.3)	*reference*	*reference*
	**Other**	26 (12.5)	22 (84.6)	4 (15.5)	1.47 (0.73; 2.96)	2.44 (0.53; 11.28)	180 (94.7)	10 (5.3)	2.51 (1.43; 4.40)	5.70 (0.23; 140.57)

## Data Availability

The data generated and analyzed during the current study are not publicly available, since they were purposively collected by the authors for the present study, but they are available from the corresponding author on reasonable request.

## Supplementary Materials

The following supporting information can be downloaded at: https://www.mdpi.com/article/10.3390/vaccines11121769/s1, Survey Questionnaire and Supplementary Files: Table S1. Socio-demographic profile of respondents by research centre. Number (N) and column percentage (%); Table S2. Number of doses of COVID-19 vaccine over time received by the study population (N = 5432); Table S3. Distribution of symptoms in health care workers (HCWs); Figure S1. COVID-19 vaccine uptake over time in health care workers from all four centres combined (N = 5432), by number of doses received; Figure S2. Any symptoms reported by health care workers during acute COVID-19, primary versus second infection; Figure S3. Percentage of symptoms (persisting or novel) reported by health care workers (HCWs) after first negative swab for primary COVID-19 episode.
