# Supplementary material for: A Multi-Center Study Investigating Long COVID-19 in Healthcare Workers from North-Eastern Italy: Prevalence, Risk Factors and the Impact of Pre-Existing Humoral Immunity—ORCHESTRA Project
Source: Vaccines (Basel). 2023 Nov 27;11(12):1769. doi: 10.3390/vaccines11121769 (PMC10747427; doi:10.3390/vaccines11121769)
Supplement: Supplementary file 1 [file vaccines-11-01769-s001.zip › vaccines-2715215-supplementary.pdf]

Supplementary Table S1. Socio-demographic profile of respondents by research centre. Number (N) and column percentage (%).

TERM	STRATA	VERONA (N=2.996)	PADUA (N=1,206)	TRIESTE (N=983)	MODENA-REGGIO EMILIA (N=247)
Sex	Males	716 (23.9)	227 (18.8)	271 (27.6)	96 (38.9)
	Females	2,280 (76.1)	979 (81.2)	712 (72.4)	151 (61.1)
Age	<40	932 (38.4)	307 (26.1)	262 (32.7)	215 (98.2)
	40-54	845 (34.8)	472 (41.0)	35 (41.8)	3 (1.4)
	55+	653 (26.9)	373 (32.4)	204 (25.5)	1 (0.5)
Country of birth	Italy	2,865 (96.4)	1,161 (96.4)	923 (94.3)	236 (95.9)
	Other EU country	57 (1.9)	24 (2.0)	21 (2.2)	4 (1.6)
	Extra-EU country	49 (1.7)	19 (1.6)	35 (3.6)	6 (2.4)
Ethnicity	Caucasian	2,421 (90.3)	848 (88.4)	791 (88.4)	235 (95.9)
	Non-Caucasian	260 (9.7)	160 (15.9)	104 (11.6)	10 (4.1)
Education	Junior secondary	176 (6.1)	101 (8.6)	49 (5.1)	0
	Secondary	932 (32.2)	349 (29.8)	231 (24.0)	0
	University	1,165 (40.2)	492 (42.0)	384 (39.9)	201 (81.7)
	Postgraduate	626 (21.6)	229 (19.6)	299 (31.1)	45 (18.3)
Marital status	Single	951 (32.7)	281 (24.0)	254 (26.3)	150 (62.5)
	Married	1,379 (47.5)	623 (53.3)	434 (44.9)	30 (12.5)
	Cohabitant	363 (12.5)	144 (12.3)	146 (15.1)	58 (24.2)
	Divorced / separated / widow	213 (7.3)	121 (10.4)	133 (13.8)	2 (0.8)
Job task	Medical consultant	330 (11.2)	159 (13.2)	186 (19.1)	6 (2.5)
	Medical trainee	233 (7.9)	53 (4.4)	16 (1.6)	234 (95.5)
	Nurse	958 (32.4)	583 (48.5)	348 (35.7)	0
	Laboratorist	108 (3.7)	35 (2.9)	15 (1.5)	0
	Nurse aid	277 (9.4)	160 (13.3)	90 (9.2)	0
	Administrative clerk	339 (11.5)	97 (8.1)	83 (8.5)	0
	Health technician	146 (4.9)	53 (4.4)	64 (6.6)	0
	Midwife	25 (0.9)	7 (0.6)	12 (1.2)	0
	Pharmacist	17 (0.6)	6 (0.5)	8 (0.8)	3 (1.2)
	Psychologist	15 (0.5)	8 (0.7)	30 (3.1)	0
	Physio-therapist	47 (1.6)	8 (0.7)	49 (5.0)	0
	Undergraduate student	228 (7.7)	0	0	0
	Other	235 (7.9)	33 (2.8)	73 (7.5)	2 (0.8)

Supplementary Table S2. Number of doses of COVID-19 vaccine over time received by the study population (N=5,432).

DATE	0 doses	1 dose	2 doses	3 doses	4 doses
**1 Jan 2021**	4439	656	337	0	0
**15 Jan 2021**	3498	1603	331	0	0
**1 Feb 2021**	2874	1003	1555	0	0
**14 Feb 2021**	2633	1122	1677	0	0
**1 Mar 2021**	1705	907	2820	0	0
**15 Mar 2021**	1657	859	2916	0	0
**1 Apr 2021**	1507	825	3100	0	0
**15 Apr 2021**	1470	836	3126	0	0
**1 May 2021**	1507	825	3100	0	0
**15 May 2021**	1470	836	3126	0	0
**1 Jun 2021**	1398	775	3259	0	0
**15 Jun 2021**	1380	772	3280	0	0
**1 Jul 2021**	1338	725	3369	0	0
**15 Jul 2021**	1334	721	3377	0	0
**1 Aug 2021**	1317	695	3410	10	0
**15 Aug 2021**	1298	711	3413	10	0
**1 Sep 2021**	1293	666	3422	51	0
**15 Sep 2021**	1289	659	3428	56	0
**1 Oct 2021**	1141	600	3258	433	0
**15 Oct 2021**	1229	583	3056	563	1
**1 Nov 2021**	1160	479	2219	1573	1
**15 Nov 2021**	1147	450	1886	1948	1
**1 Dec 2021**	1093	347	1123	2868	1
**15 Dec 2021**	1077	307	780	3267	1
**1 Jan 2022**	1064	272	631	3463	2
**15 Jan 2022**	1052	263	612	3502	3
**1 Feb 2022**	1047	245	571	3566	3
**14Feb 2022**	1046	241	567	3575	3
**1 Mar 2022**	1037	240	533	3618	4
**15 Mar 2022**	1037	234	526	3631	4
**1 Apr 2022**	1034	229	499	3664	6
**15 Apr 2022**	1035	224	497	3669	7
**1 May 2022**	1029	224	456	3715	8
**15 May 2022**	1028	224	451	3721	8
**1 Jun 2022**	1026	219	420	3758	9
**15 Jun 2022**	1025	217	417	3764	9
**1 Jul 2022**	1023	214	410	3775	10
**15 Jul 2022**	1022	212	410	3777	11
**1 Aug 2022**	1021	212	410	3777	12
**15 Aug 2022**	1021	212	410	3777	12
**1 Sep 2022**	1021	212	408	3779	12

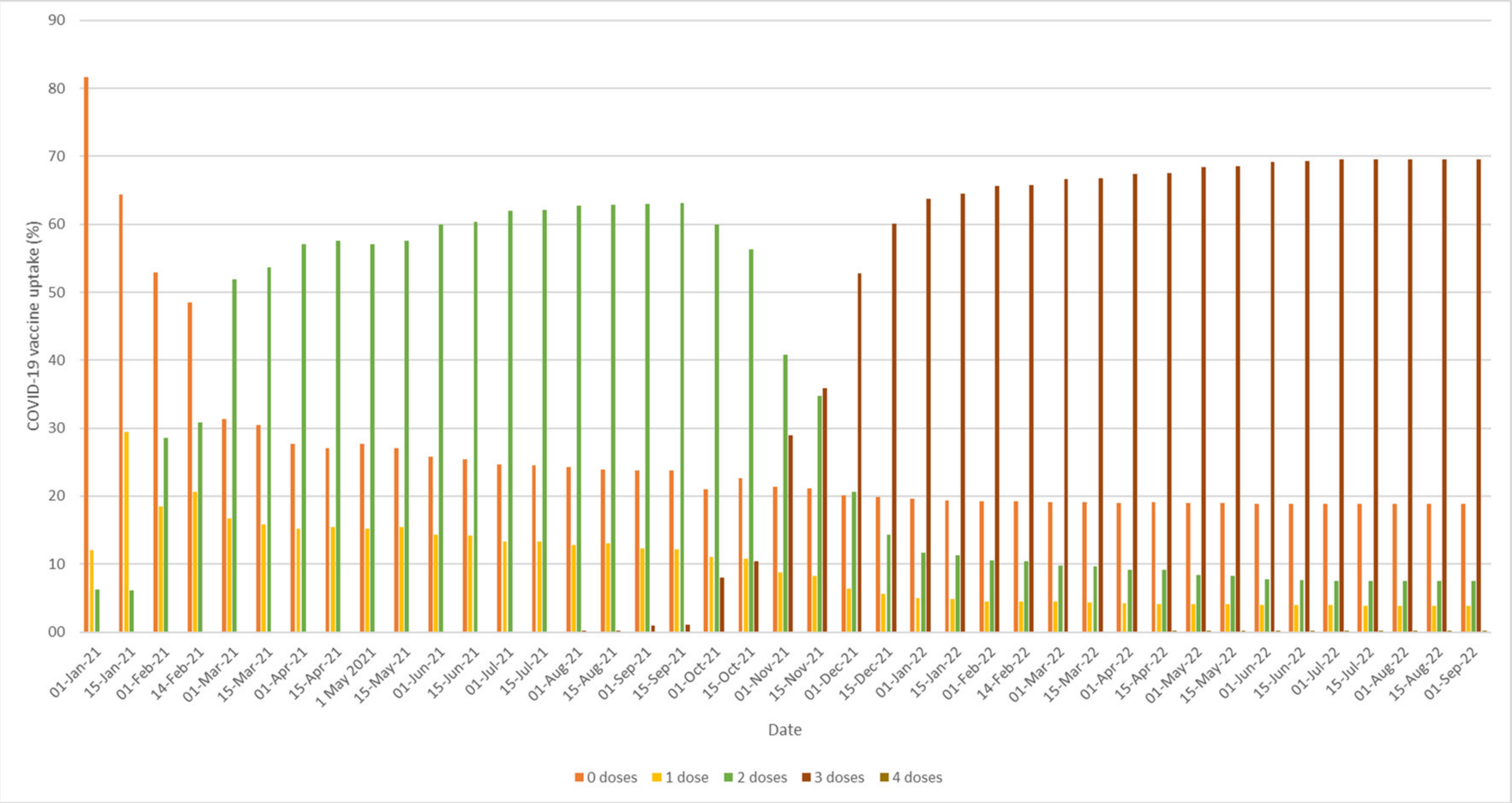

Supplementary Figure S1. COVID-19 vaccine uptake over time in health care workers from all four centres combined (N=5,432), by number of doses received.

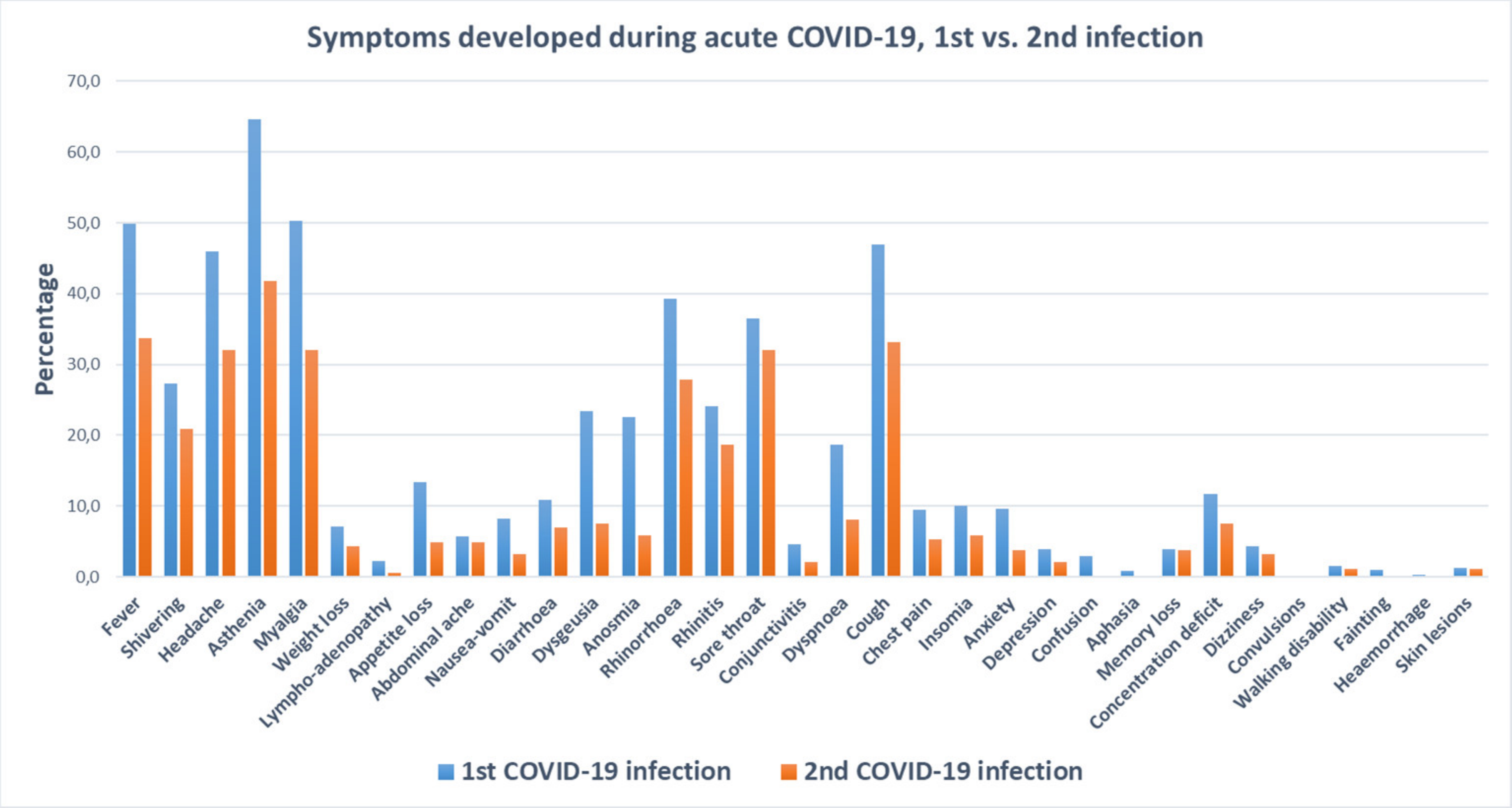

Supplementary Figure S2. Any symptoms reported by health care workers during acute COVID-19, primary versus second infection.

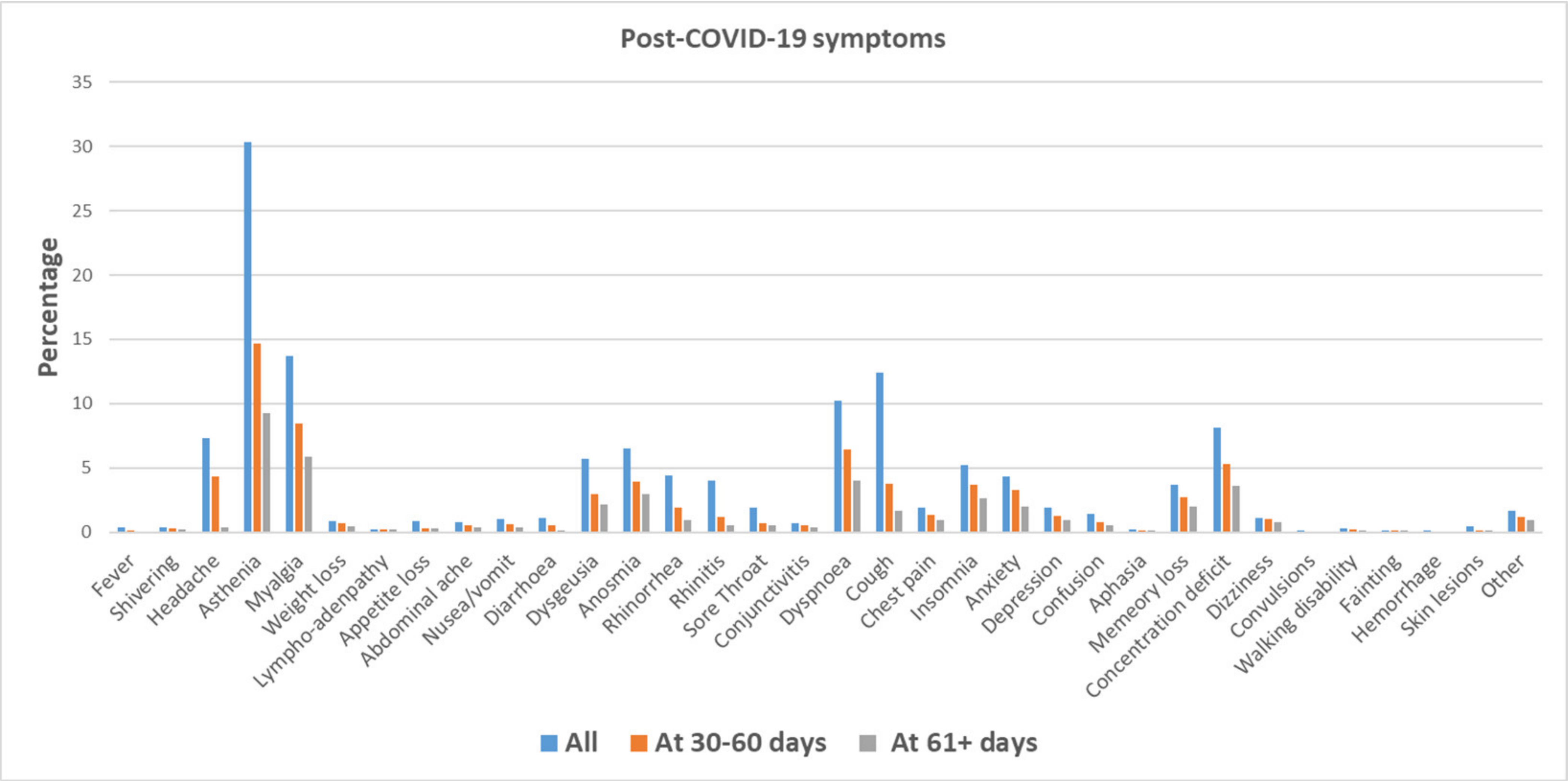

Supplementary Figure S3. Percentage of symptoms (persisting or novel) reported by health care workers (HCWs) after first negative swab for primary COVID-19 episode. Overall symptoms, at 30-60 days or 61+ days since first negative swab following primary COVID-19 infection

Supplementary Table S3. Distribution of symptoms in health care workers (HCWs). Symptoms during acute disease, all post-COVID-19 symptoms, persisting or newly developed at 30-60 or 61+ days since first negative swab (primary vs. second infection). Number (N) and column percentage (%). ENT= Ear-Nose-Throat. NA= Not applicable. Symptoms not available on 432 HCWs for 1st COVID-19 infection and on 51 HCWs for second COVID-19 episode. M=missing information on symptoms

SYMPTOMS		STRATA	SYMPTOMS RELATED TO 1st COVID-19 INFECTION (M=432))							SYMPTOMS RELATED TO 2nd COVID-19 INFECTION (M=51)				
			ACUTE	All Post COVID-19	At 30-60 days (N=576)			At 61+ days (N=389)		ACUTE	All Post COVID-19	At 30-60 days (N=25)		
					ALL	PERSISTING	NOVEL	PERSISTING	NOVEL			ALL	PERSISTING	NOVEL
GENERAL INFECTIOUS	Fever	No	987 (50.1)	1,961 (99.6)	453 (99.6)	289 (99.3)	164 (100)	189 (99.5)	108 (100)	124 (66.3)	187 (100)	19 (100)	6 (100)	13 (100)
		Yes	982 (49.9)	8 (0.4)	2 (0.4)	2 (0.7)	0	1 (0.5)	0	63 (26.5)	0	0	0	0
	Shivering	No	1,431 (72.7)	1,862 (99.6)	449 (98.7)	164 (96.5)	285(100)	115 (95.8)	178 (100)	148 (79.1)	146 (78.1)	5 (26.3)	1 (25.0)	4 (26.7)
		Yes	538 (27.3)	7 (0.4)	6 (1.3)	6 (3.5)	0	5	0	39 (20.9)	41 (21.9)	14 (73.7)	3 75.0)	11 (73.3)
	Headache	No	1,064 (54.0)	1,826 (92.7)	370 (81.4)	210 (72.7)	160 (96.4)	142 (73.6)	102 (97.1)	127 (67.9)	182 (97.3)	17 (89.5)	7 (77.8)	10 (100)
		Yes	905 (46.0)	143 (7.3)	85 (18.6)	79 (27.3)	6 (3.6)	51 (26.4)	3 (2.9)	60 (32.1)	5 (2.7)	2 (10.5)	2 (2.2)	0
	Asthenia	No	696 (35.4)	1,373 (69.7)	166 (36.6)	119 (30.7)	47 (70.2)	86 (33.3)	29 (72.5)	109 (58.3)	162 (86.6)	11 (57.9)	8 (57.1)	3 (60.8)
		Yes	1,273 (64.7)	596 (30.3)	289 (63.4)	269 (69.3)	20 (27.5)	172 (66.7)	11 (27.5)	78 (41.7)	25 (13.4)	8 (42.1)	6 (42.9)	2 (40.0)
	Myalgia	No	980 (49.8)	1699 (86.3)	288 (63.4)	177 (54.1)	111 (86.7)	113 (52.1)	69 (85.2)	127 (67.9)	187 (100)	19 (100)	10 (100)	9 (100)
		Yes	989 (50.2)	270 (13.7)	167 (36.6)	150 (45.9)	17 (13.3)	104 (47.9)	12 (14.8)	60 (32.1)	0	0	0	0
	Weight loss	No	1,830 (92.9)	1,952 (99.1)	441 (96.9)	63 (84.0)	378 (99.5)	51 (87.9)	238 (99.2)	179 (95.7)	187 (100)	19 (100)	19 (100)	19 (100)
		Yes	139 (7.1)	17 (0.9)	14 (3.1)	12 (16.0)	2 (0.5)	7 (12.1)	2 (0.8)	8 (4.3)	0	0	0	0
	Lympho-adenopathy	No	1,924 (97.7)	1,964 (99.8)	451 (99.1)	14 (77.8)	437 (100)	11 (73.3)	283 (100)	186 (99.5)	187 (100)	19 (100)	19 (100)	19 (100)
		Yes	45 (2.3)	5 (0.2)	4 (0.9)	4 (22.2)	0	4 (26.7)	0	1 (0.5)	0	0	0	0
GASTRO-ENETERIC	Appetite loss	No	1,705 (86.6)	1,951 (99.1)	449 (98.7)	106 (95.5)	343 (99.7)	74 (93.7)	218 (99.5)	178 (95.2)	177 (94.7)	16 (84.2)	1 (50)	15 (88.2)
		Yes	264 (13.4)	18 (0.9)	6 (1.3)	5 (4.5)	1 (0.3)	5 (6.3)	1 (0.5)	9 (4.8)	10 (5.4)	3 (15.8)	1 (50)	2 (11.8)
	Abdominal ache	No	1,857 (94.3)	1,954 (99.2)	444 (97.6)	50 (83.3)	394 (99.8)	39 (83)	251 (100)	178 (95.2)	185 (98.9)	19 (100)	2 (100)	17 (100)
		Yes	112 (5.7)	15 (0.8)	11 (2.4)	10 (16.7)	1 (0.2)	8 (17)	0	9 (4.8)	2 (1.1)	0	0	0
	Nausea/ vomit	No	1,807 (91.8)	1,950 (99.0)	443 (97.4)	56 (84.6)	387 (99.5)	39 (86.7)	251 (99.2)	181 (96.8)	185 (98.9)	18 (94.7)	0	18 (94.7)
		Yes	162 (8.2)	19 (1.0)	12 (2.6)	10 (15.2)	2 (0.5)	6 (13.3)	2 (0.8)	6 (3.2)	2 (1.1)	1 (5.3)	0	1 (5.3)
	Diarrhoea	No	1,755 (89.1)	1,948 (98.9)	445 (97.8)	81 (90.0)	364 (99.7)	62 (96.9)	233 (99.6)	174 (93.1)	184 (98.4)	19 (100)	1 (100)	18 (100)
		Yes	214 (10.9)	21 (1.1)	10 (2.2)	9 (10.0)	1 (0.3)	2 (3.1)	1 (0.4)	13 (7.0)	3 (1.6)	0	0	0
ENT	Dysgeusia	No	1,508 (76.6)	1,856 (94.3)	396 (87.0)	147 (73.5)	249 (97.7)	107 (74.3)	149 (96.8)	173 (92.5)	182 (97.3)	17 (89.5)	3 (75)	14 (93.3)
		Yes	461 (23.4)	113 (5.7)	59 (13.0)	53 (26.5)	6 (2.4)	37 (25.7)	5 (3.3)	14 (7.5)	5 (2.7)	2 (10.5)	1 (25)	1 (6.7)
	Anosmia	No	1,525 (77.5)	1,841 (93.5)	378 (83.1)	121 (62.1)	257 (98.6)	85 (60.3)	155 (98.7)	176 (94.1)	180 (96.3)	18 (94.7)	3 (100)	15 (93.8)
		Yes	444 (22.6)	128 (6.5)	77 (16.9)	74 (38.0)	3 (1.2)	56 (39.7)	2 (1.3)	11 (5.9)	7 (3.7)	1 (5.3)	0	1 (6.3)
	Rhinorrhoea	No	1,196 (60.7)	1,882 (95.6)	418 (91.9)	156 (83.0)	262 (98.1)	99 (86.1)	181 (98.9)	135 (72.2)	184 (98.4)	18 (94.7)	4 (80)	14 (100)
		Yes	773 (39.3)	87 (4.4)	37 (8.1)	32 (17.0)	5 (1.9)	16 (13.9)	2 (1.1)	52 (27.8)	3 (1.6)	2 (5.3)	1 (20)	0
	Rhinitis	No	1,496 (76.0)	1,890 (96.0)	431 (94.7)	89 (84.8)	342 (97.7)	56 (91.8)	231 (97.5)	152 (81.2)	173 (92.5)	16 (84.2)	2 (66.7)	14 (87.5)
		Yes	473 (24.0)	79 (4.0)	24 (5.3)	16 (15.2)	8 (2.3)	5 (8.2)	6 (2.5)	35 (18.7)	14 (5.9)	3 (15.8)	1 (33.3)	2 (12.5)
	Sore throat	No	1,251 (63.5)	1,931 (98.1)	441 (96.9)	175 (93.6)	266 (99.3)	105 (92.1)	182 (98.9)	127 (67.9)	186 (99.5)	19 (100)	3 (100)	16 (84.2)
		Yes	718 (36.5)	38 (1.9)	14 (3.1)	12 (6.4)	2 (0.7)	9 (7.9)	2 (1.1)	60 (32.1)	1 (0.5)	0	0	3 (15.8)
	Conjunctivitis	No	1,879 (95.4)	1,955 (99.3)	445 (97.8)	27 (73.0)	418 (100)	15 (68.2)	276 (100)	183 (97.9)	187 (100)	19 (100)	1 (100)	18 (100)
		Yes	90 (4.6)	14 (0.7)	10 (2.2)	10 (27.0)	0	7 (31.8)	0	4 (2.1)	0	0	0	0

**PULMONARY**	**Dyspnoea**	**No**	1,601 (81.3)	1,769 (89.8)	328 (72.2)	88 (44.7)	240 (93.0)	68 (49.6)	151 (93.8)	172 (92.0)	169 (90.4)	16 (84.2)	2 (50)	14 (93.3)
		**Yes**	368 (18.7)	200 (10.2)	127 (27.9)	109 (55.3)	18 (7.0)	69 (5.4)	10 (6.2)	15 (8.0)	18 (9.6)	3 (15.8)	2 (50)	1 (6.7)
	**Cough**	**No**	1,046 (53.1)	1,725 (87.6)	381 (83.8)	180 (71.7)	201 (98.5)	137 (80.6)	128 (100)	125 (66.8)	185 (98.9)	18 (94.7)	5 (83.3)	13 (100)
		**Yes**	923 (46.9)	244 (12.4)	74 (16.2)	71 (28.3)	3 (1.5)	33 (19.4)	0	62 (33.2)	2 (1.1)	1 (5.3)	1 (16.7)	0
	**Chest pain**	**No**	1,782 (90.5)	1,931 (98.1)	429 (94.3)	70 (75.3)	359 (99.2)	51 (75)	229 (99.6)	177 (94.7)	175 (93.6)	15 (79.9)	2 (50)	13 (86.7)
		**Yes**	187 (9.5)	38 (1.9)	26 (5.7)	23 (24.7)	3 (0.8)	17 (25)	1 (0.4)	10 (5.3)	12 (5.0)	4 (21.1)	2 (50)	2 (13.3)
**PSYCHOLOGICAL**	**Insomnia**	**No**	1,771 (89.9)	1,867 (94.8)	382 (84.0)	49 (46.7)	333 (95.1)	36 (46.7)	210 (95.0)	176 (94.1)	182 (97.3)	17 (89.5)	3 (75)	14 (93.3)
		**Yes**	198 (10.1)	102 (5.2)	73 (16.0)	56 (53.3)	17 (4.9)	41 (53.2)	11 (5.0)	11 (5.9)	5 (2.7)	2 (10.5)	1 (25)	1 (6.7)
	**Anxiety**	**No**	1,781 (90.5)	1,885 (95.7)	391 (86.0)	58 (52.3)	333 (96.4)	44 (57.1)	214 (96.8)	180 (96.3)	184 (98.4)	19 (100)	3 (100)	16 (100)
		**Yes**	188 (9.5)	84 (4.3)	64 (14.0)	53 (47.3)	11 (3.2)	33 (42.9)	7 (3.2)	7 (3.7)	3 (1.6)	0	0	0
	**Depression**	**No**	1,893 (96.1)	1,932 (98.1)	430 (94.5)	28 (63.6)	523 (97.8)	23 (63.9)	256 (97.7)	183 (97.9)	187 (100)	19 (100)	0	19 (100)
		**Yes**	76 (3.9)	38 (1.9)	25 (5.5)	16 (36.4)	9 (2.2)	13 (36.1)	6 (2.3)	4 (2.1)	0	0	0	0
**NEUROLOGICAL**	**Confusion**	**No**	1,912 (97.1)	1,942 (98.6)	439 (96.5)	22 (75.9)	417 (97.8)	18 (78.3)	269 (97.8)	185 (98.9)	187 (100)	19 (100)	0	19 (100)
		**Yes**	57 (2.9)	27 (1.4)	16 (3.5)	7 (24.1)	9 (2.1)	5 (21.7)	6 (2.2)	2 (1.1)	0	0	0	0
	**Aphasia**	**No**	1,953 (99.2)	1,965 (99.8)	453 (99.6)	4 (80.0)	449 (99.8)	2 (66.7)	294 (99.7)	187 (100)	179 (95.7)	17 (89.5)	0	17 (89.5)
		**Yes**	16 (0.8)	4 (0.2)	2 (0.4)	1 (20.0)	1 (0.2)	1 (33.3)	1 (0.3)	0	8 (4.3)	2 (1.5)	0	2 (10.5)
	**Memory loss**	**No**	1,892 (96.1)	1,897 (96.3)	401 (88.2)	26 (44.8)	375 (94.5)	19 (44.2)	240 (94.1)	180 (96.3)	170 (90.9)	13 (58.4)	1 (100)	12 (66.7)
		**Yes**	77 (3.9)	72 (3.7)	54 (11.8)	32 (55.2)	22 (5.5)	24 (55.8)	15 (5.9)	7 (3.7)	17 (9.1)	6 (31.6)	0	6 (33.3)
	**Concentration deficit**	**No**	1,740 (88.4)	1,809 (91.9)	351 (77.2)	52 (39.1)	299 (92.9)	35 (37.6)	192 (93.7)	173 (92.5)	185 (98.9)	19 (100)	4 (100)	15 (100)
		**Yes**	229 (11.6)	160 (8.1)	104 (22.8)	81 (60.9)	23 (7.1)	58 (62.4)	13 (6.3)	14 (7.5)	2 (1.1)	0	0	0
	**Dizziness**	**No**	1,883 (95.6)	1,947 (98.9)	435 (95.6)	34 (68.0)	401 (99.0)	26 (66.7)	256 (98.8)	181 (96.8)	187 (100)	19 (100)	0	19 (100)
		**Yes**	86 (4.4)	22 (1.1)	20 (4.4)	16 (32.0)	4 (1.0)	13 (33.3)	3 (1.2)	6 3.2)	0	0	0	0
	**Convulsions**	**No**	1,968 (100)	1,968 (99.9)	454 (99.8)	0	454 (99.8)	0	297 (99.7)	187 (100)	186 (99.5)	19 (100)	0	19 (100)
		**Yes**	1	1 (0.1)	1 (0.2)	0	1 (0.2)	0	1 (0.3)	0	1 (0.5)	0	0	0
	**Walking disability**	**No**	1,940 (98.5)	1,963 (99.7)	451 (99.1)	18 (94.7)	433 (99.3)	14 (93.3)	281 (99.3)	185 (98.9)	187 (100)	19 (100)	1 (100)	18 (100)
		**Yes**	29 (1.5)	6 (0.3)	4 (0.9)	1 (5.3)	3 (0.7)	1 (6.7)	2 (0.7)	2 (1.1)	0	0	0	0
	**Fainting**	**No**	1,949 (99)	1,966 (99.9)	452 (99.3)	12 (100)	440 (99.3)	10 (100)	286 (99.3)	187 (100)	187 (100)	19 (100)	0	19 (100)
		**Yes**	20 (1)	3 (0.1)	3 (0.7)	0	3 (0.7)	0	2 (0.7)	0	0	0	0	0
**OTHER**	**Haemorrhage**	**No**	1.964 (99.7)	1,968 (99.9)	454 (99.8)	2 (66.7)	452 (100)	2 (100)	296 (100)	187 (100)	186 (99.5)	19 (100)	0	19 (100)
		**Yes**	5 (0.3)	1 (0.1)	1 (0.2)	1 (33.3)	0	0	0	0	1 (0.5)	0	0	0
	**Skin lesions**	**No**	1,943 (98.7)	1,960 (99.5)	452 (99.3)	7 (70.0)	445 (100)	6 (66.7)	289 (100)	185 (98.9)	186 (99.5)	19 (100)	0	19 (100)
		**Yes**	26 (1.3)	9 (0.5)	3 (0.7)	3 (30.0)	0	3 (33.3)	0	2 (1.1)	1 (0.5)	0	0	0
	**Other**	**No**	NA	1,935 (98.3)	431 (94.7)	NA	431 (94.7)	NA	279 (93.6)	NA	187 (100)	19 (100)	NA	19 (100)
		**Yes**	NA	34 (1.7)	24 (5.3)	NA	24 (5.3)	NA	19 (6.4)	NA	0	0	NA	0
